# An IL-6/STAT3/MR/FGF21 axis mediates heart-liver cross-talk after myocardial infarction

**DOI:** 10.1126/sciadv.ade4110

**Published:** 2023-04-05

**Authors:** Jian-Yong Sun, Lin-Juan Du, Xue-Rui Shi, Yu-Yao Zhang, Yuan Liu, Yong-Li Wang, Bo-Yan Chen, Ting Liu, Hong Zhu, Yan Liu, Cheng-Chao Ruan, Zhenji Gan, Hao Ying, Zhinan Yin, Ping-Jin Gao, Xiaoxiang Yan, Ruo-Gu Li, Sheng-Zhong Duan

**Affiliations:** ^1^ Laboratory of Oral Microbiota and Systemic Diseases, Shanghai Ninth People’s Hospital, College of Stomatology, Shanghai Jiao Tong University School of Medicine, Shanghai 200125, China.; ^2^ National Center for Stomatology; National Clinical Research Center for Oral Diseases; Shanghai Key Laboratory of Stomatology, Shanghai 200011, China.; ^3^ Department of Cardiology, Shanghai Chest Hospital, Shanghai Jiao Tong University, Shanghai 200030, China.; ^4^ Department of Medicine, Diabetes Unit and Center for Genomic Medicine, Massachusetts General Hospital, Boston, MA 02114, USA.; ^5^ Department of Physiology and Pathophysiology, School of Basic Medical Sciences, Fudan University, Shanghai, China.; ^6^ State Key Laboratory of Pharmaceutical Biotechnology and MOE Key Laboratory of Model Animal for Disease Study, Jiangsu Key Laboratory of Molecular Medicine, Chemistry and Biomedicine Innovation Center (ChemBIC), Model Animal Research Center, Nanjing University Medical School, Nanjing University, Nanjing 210061, China.; ^7^ CAS Key Laboratory of Nutrition, Metabolism, and Food Safety, Shanghai Institute of Nutrition and Health, Chinese Academy of Sciences, Shanghai 200031, China.; ^8^ Guangdong Provincial Key Laboratory of Tumor Interventional Diagnosis and Treatment, Zhuhai Institute of Translational Medicine Zhuhai People’s Hospital Affiliated with Jinan University, Jinan University, Zhuhai 519000, Guangdong, China.; ^9^ The Biomedical Translational Research Institute, Health Science Center (School of Medicine), Jinan University, Guangzhou 510632, Guangdong, China.; ^10^ Department of Cardiovascular Medicine, State Key Laboratory of Medical Genomics, Shanghai Key Laboratory of Hypertension, Shanghai Institute of Hypertension, Ruijin Hospital, Shanghai Jiao Tong University School of Medicine, Shanghai 200025, China.; ^11^ Department of Cardiovascular Medicine, Ruijin Hospital, Shanghai Jiao Tong University School of Medicine, Shanghai 200025, China.; ^12^ Shanghai Key Laboratory of Hypertension, Shanghai Institute of Hypertension, Shanghai, China.

## Abstract

The liver plays a protective role in myocardial infarction (MI). However, very little is known about the mechanisms. Here, we identify mineralocorticoid receptor (MR) as a pivotal nexus that conveys communications between the liver and the heart during MI. Hepatocyte *MR* deficiency and MR antagonist spironolactone both improve cardiac repair after MI through regulation on hepatic fibroblast growth factor 21 (FGF21), illustrating an MR/FGF21 axis that underlies the liver-to-heart protection against MI. In addition, an upstreaming acute interleukin-6 (IL-6)/signal transducer and activator of transcription 3 (STAT3) pathway transmits the heart-to-liver signal to suppress MR expression after MI. Hepatocyte *Il6* receptor deficiency and *Stat3* deficiency both aggravate cardiac injury through their regulation on the MR/FGF21 axis. Therefore, we have unveiled an IL-6/STAT3/MR/FGF21 signaling axis that mediates heart-liver cross-talk during MI. Targeting the signaling axis and the cross-talk could provide new strategies to treat MI and heart failure.

## Teaser

Activation of communications between the heart and the liver protects the heart from myocardial infarction.

## INTRODUCTION

Interorgan communication is crucial for the coordination of organ functions, maintenance of homeostasis, and disease adaptation (*1*, *2*). For example, exercise has shown cardioprotection through communication from the skeletal muscle to the heart (*3*). Previous clinical reports have suggested that normal liver function has protective effects on lungs and is essential for the recovery of lungs after injuries (*4*, *5*). Retrospective clinical studies have shown that kidney transplantation notably improves cardiac structure and function of patients with end-stage renal disease, implying a protective role of kidney-heart communication in cardiovascular diseases (*6*, *7*). However, very little is understood about the mechanistic underpinnings of organ communications.

Myocardial infarction (MI) remains the leading cause of death among cardiovascular diseases (*8*). The liver has been implied to play a protective role in the pathologic process of MI through up-regulating and secreting cardioprotective proteins, including fibroblast growth factor 21 (FGF21), bone morphogenetic protein–binding endothelial regulator, and α-1-acid glycoprotein type 2 (*9*, *10*). However, the mechanisms underlying the hepatic protective effects are poorly appreciated, the key regulators that promote the expression and secretion of hepatokines after MI remain elusive, and the upstream signals that may mediate the communication from the heart to the liver are largely unknown.

Nuclear receptors are a group of transcription factors that play critical roles in development, reproduction, homeostasis, and various diseases including cardiovascular diseases (*11*, *12*). Activation of peroxisome proliferator–activated receptors (*PPARs*), including *PPAR*α, *PPAR*β*/*δ, and *PPAR*γ, has been shown to exert beneficial effects in cardiovascular diseases such as atherosclerosis and MI (*13*, *14*). Overactivation of nuclear receptor subfamily 3 group C member 2 (*Nr3c2*; also known as mineralocorticoid receptor or *MR*) aggravates cardiovascular diseases, including hypertension, atherosclerosis, and MI (*15*–*17*). Activation of nuclear receptor subfamily 1 group D members 1 and 2 (*Nr1d1* and *Nr1d2*; also known as *REV-ERB*α and *REV-ERB*β) reduces atherosclerotic plaques and improves adverse cardiac remodeling after MI (*18*–*20*). Nuclear receptors are highly druggable targets (*21*). For example, PPARα agonists, PPARγ agonists, and MR antagonists are all clinically approved and commonly used to treat patients with metabolic and/or cardiovascular diseases (*16*, *21*). It is of great interest to explore the function of transcription factors, particularly nuclear receptors, in the cross-talk between the heart and the liver in the process of cardiovascular diseases.

In this context, we performed RNA sequencing (RNA-seq) to search for the markedly altered nuclear receptors in the liver after MI and observed substantial decrease of *MR*. We then assessed the impacts of hepatocyte *MR* deficiency on MI in mice. Subsequently, we found that FGF21 was the most notably increased secretory protein that mediated the effects of hepatocyte *MR* deficiency and spironolactone in post-MI mice and/or patients with heart failure (HF) and dissected the regulatory mechanisms of *MR* on *Fgf21*. Last, we went further to uncover the upstream mechanism that regulated hepatic *MR* to mediate the communication from the heart to the liver after MI.

## RESULTS

### Deficiency of hepatocyte *MR* improves cardiac function and decreases infarct scar size after acute or subacute MI in mice

**Fig. 1.**
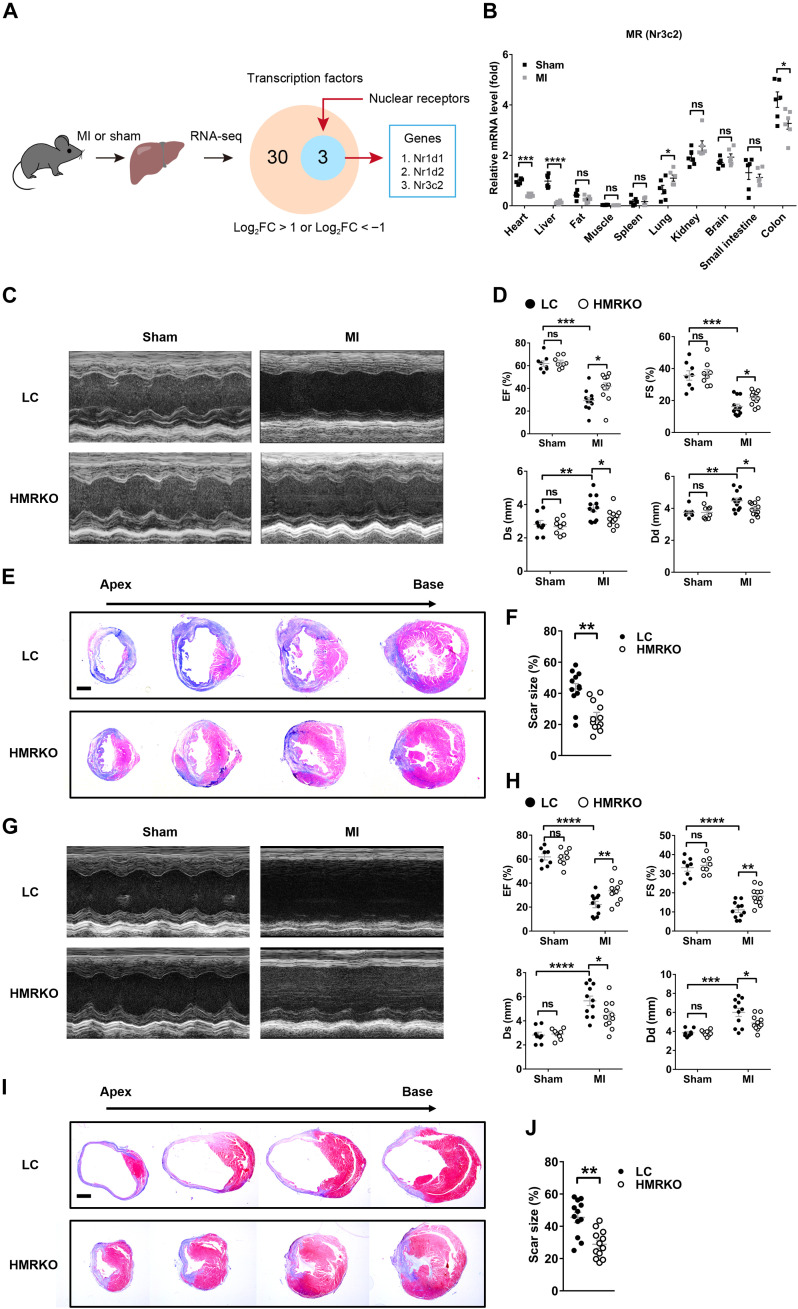
Deficiency of hepatocyte *MR* improves cardiac function and decreases scar size after acute or subacute MI in mice. (**A**) Schematic illustration of the experimental approach to identify significantly altered nuclear receptors in the liver responding to MI. The Venn diagram showed differentially expressed transcription factors and nuclear receptors in livers 1 day after MI versus sham operation. FC, fold change; *Nr1d1* and *Nr1d2*, nuclear receptor subfamily 1 group D members 1 and 2, respectively (also known as *REV-ERB*α and *REV-ERB*β); *Nr3c2*, nuclear receptor subfamily 3 group C member 2 (also known as *MR*). *n* = 3. (**B**) qRT-PCR analysis of *MR* (or *Nr3c2*) gene expression in different mouse tissues 1 day after MI. *n* = 6. (**C**) Representative images of echocardiography in littermate control (LC) and hepatocyte *MR* knockout (HMRKO) mice 1 week after MI or sham operation. (**D**) Quantifications of ejection fraction (EF), fraction shortening (FS), left ventricular end-systolic dimension (Ds), and left ventricular end-diastolic dimension (Dd) based on echocardiography. Sham, *n* = 8; MI, *n* = 12. (**E**) Representative Masson’s trichrome staining of mouse cardiac sections 1 week after MI or sham operation. Scar tissues stained blue. Scale bar, 1 mm. (**F**) Quantification of scar size in hearts exemplified in (E). *n* = 12. DAPI, 4′,6-diamidino-2-phenylindole. (**G**) Representative images of echocardiography in mice 8 weeks after MI or sham operation. (**H**) Quantifications of EF, FS, Ds, and Dd based on echocardiography. Sham, *n* = 8; MI, *n* = 11. (**I**) Representative Masson’s trichrome staining of mouse cardiac sections 8 weeks after MI or sham operation. Scale bar, 1 mm. (**J**) Quantification of scar size in hearts exemplified in (I). *n* = 12. Data are represented as means ± SEM. ns, not significant. *P < 0.05, **P < 0.01, ***P < 0.001, and ****P < 0.0001.

To determine whether nuclear receptors play important roles in heart-liver cross-talk, we performed RNA-seq analysis of mouse livers 1 day after MI, detected 518 differentially expressed genes compared to sham operation, and identified three nuclear receptors among 30 differentially expressed transcription factors (Fig. 1A and fig. S1A). All three nuclear receptors—*Nr1d1*, *Nr1d2*, and *Nr3c2* (also known as *MR*)—were markedly down-regulated in the liver after MI (Fig. 1A). Previous studies have shown that the decrease of *Nr1d1* and *Nr1d2* leads to dysfunction of circadian rhythm and aggravates cardiovascular diseases (*22*, *23*). On the contrary, the reduction of *MR* mostly plays protective roles in cardiovascular diseases (*24*–*26*). In this context, we focused on the role of *MR* in the liver after MI. Results of quantitative reverse transcription polymerase chain reaction (qRT-PCR) demonstrated that the liver manifested the most notable decrease in *MR* expression compared to the other organs 1 day after MI (Fig. 1B). In addition, the hepatic expression of *MR* reached the lowest level at 1 day after MI, after which the expression gradually recovered (fig. S1B). These results indicate that the reduction of hepatic MR is an early response of the liver after MI and may exert a protective role.

Hepatocyte *MR* knockout (HMRKO) mice were generated to investigate whether hepatic *MR* was involved in cardiac protection after MI. Results of qRT-PCR and Western blotting showed nearly nondetectable levels of MR in livers from HMRKO mice compared to littermate control (LC) mice (fig. S1, C and D). Moreover, the deficiency of *MR* was restricted to the liver of the HMRKO mice (fig. S1E). Echocardiography of HMRKO mice displayed significantly higher ejection fraction (EF) and fractional shortening (FS), as well as significantly lower left ventricular systolic dimension (Ds) and left ventricular diastolic dimension (Dd) than that of LC mice 1 week after MI (Fig. 1, C and D). Masson’s trichrome staining of cardiac sections from HMRKO mice showed nearly 50% reduction in infarct scar size at this time point (Fig. 1, E and F). Similarly, cardiac function was markedly improved and infarct scar size was significantly decreased in HMRKO mice compared to LC mice at 4 and 8 weeks after MI (Fig. 1, G to J, and fig. S1, F to I). These data together clearly demonstrated a protective role of hepatocyte *MR* deficiency in MI.

### Deficiency of hepatocyte *MR* inhibits adverse cardiac remodeling after MI in mice

**Fig. 2.**
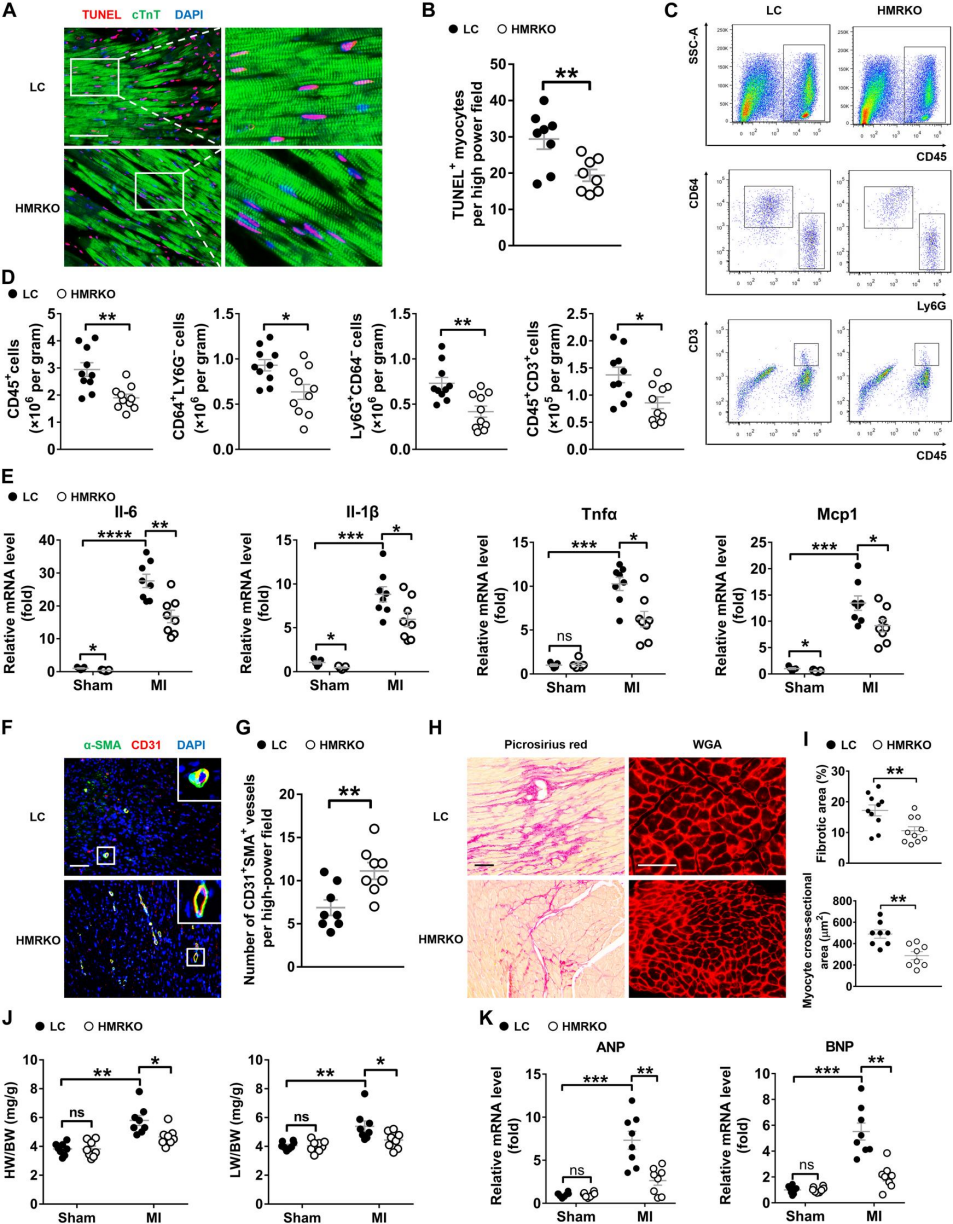
Deficiency of hepatocyte *MR* inhibits adverse cardiac remodeling after MI in mice. (**A**) Representative TUNEL staining and cardiac troponin T (cTnT) immunofluorescence staining in cardiac sections from LC and HMRKO mice 3 days after MI. (**B**) Quantification of TUNEL-positive myocytes. *n* = 8. (**C**) Representative flow cytometry analysis of CD45^+^ cells, CD64^+^Ly6G^−^ macrophages, Ly6G^+^CD64^−^ granulocytes, and CD45^+^CD3^+^ lymphocytes in mouse heart samples 3 days after MI. SSC-A, side scatter area. (**D**) Quantification of flow cytometry results. *n* = 10. (**E**) qRT-PCR analysis of proinflammatory cytokines in mouse heart samples 3 days after MI or sham operation. Sham, *n* = 5; MI, *n* = 8. (**F**) Representative immunofluorescence staining of alpha-smooth muscle actin (α-SMA) and CD31 in the peri-infarct regions of mouse heart samples 7 days after MI. (**G**) Quantification of α-SMA and CD31 dual-positive vessels in peri-infarct regions. *n* = 8. (**H**) Representative Picrosirius red staining (left) and wheat germ agglutinin (WGA) staining (right) in the noninfarct regions of mouse heart samples 8 weeks after MI. (**I**) Quantitative analyses of fibrotic area (red areas in Picrosirius red staining) and cardiomyocyte size (WGA staining). *n* = 8 to 10. (**J**) Heart weight–to–body weight ratio (HW/BW) and lung weight–to–body weight ratio (LW/BW) of mice 8 weeks after MI or sham operation. *n* = 8. (**K**) qRT-PCR analysis of *ANP* (atrial natriuretic peptide) and *BNP* (brain natriuretic peptide) gene expression in mouse heart samples 8 weeks after MI or sham operation. *n* = 8. Scale bars, 100 μm. Data are represented as means ± SEM. *P < 0.05, **P < 0.01, ***P < 0.001, and ****P < 0.0001.

Cardiac remodeling after MI involves several different processes, including death of cardiomyocytes, cardiac inflammation, neovascularization, cardiac fibrosis, cardiac hypertrophy, and possibly chamber dilation (HF), all of which have significant impacts on the outcome of the myocardial repair (*27*). Terminal deoxynucleotidyl transferase–mediated deoxyuridine triphosphate nick end labeling (TUNEL) staining showed a significant reduction of apoptotic cardiomyocytes in the border region of hearts from HMRKO mice compared to LC mice 3 days after MI (Fig. 2, A and B). Consistently, HMRKO markedly decreased the level of cleaved caspase-3 in hearts after MI (fig. S2, A and B). Flow cytometry results revealed that MI-induced infiltration of CD45^+^ cells, CD64^+^Ly6G^−^ macrophages, Ly6G^+^CD64^−^ granulocytes, and CD45^+^CD3^+^ lymphocytes were considerably diminished in hearts from HMRKO mice compared to LC mice (Fig. 2, C and D, and fig. S2C). Accordingly, qRT-PCR analysis demonstrated that the expression of proinflammatory cytokines, including interleukin-6 (*Il6*), *Il1*β, tumor necrosis factor–α (*Tnf*α), and monocyte chemoattractant protein-1 (*Mcp1*), were notably decreased in HMRKO mouse hearts after MI (Fig. 2E). New blood vessel formation is necessary for recovery of tissue function after ischemia or tissue injury. Immunofluorescence staining revealed that HMRKO increased the number of blood vessels in peri-infarct regions, remote regions, and infarct regions 7 days after MI (Fig. 2, F and G, and fig. S2, D and E).

Fibrosis area and cardiomyocyte size in the noninfarct region of HMRKO mouse hearts were notably smaller than those of LC mice 8 weeks after MI (Fig. 2, H and I). Consistently, the expression of fibrogenic genes was substantially decreased in the noninfarct region of HMRKO mouse hearts after MI (fig. S2F). HMRKO mice also had significantly smaller heart weight–to–body weight ratio (HW/BW), heart weight to tibal lengh ratio (HW/TL), and lung weight–to–body weight ratio (LW/BW) than LC mice, suggesting mitigated cardiac hypertrophy and HF (Fig. 2J and fig. S2G). Consistently, the MI-elevated expression of HF markers such as atrial natriuretic peptide (*ANP*) and brain natriuretic peptide (*BNP*) was markedly decreased in the hearts of HMRKO mice (Fig. 2K). Together, the deficiency of hepatocyte *MR* inhibited post-MI adverse cardiac remodeling, reflected by reduced apoptosis of cardiomyocytes, decreased inflammation, increased angiogenesis, as well as alleviated cardiac fibrosis, hypertrophy, and HF.

### FGF21 mediates the protection of hepatocyte MR deficiency on MI

**Fig. 3.**
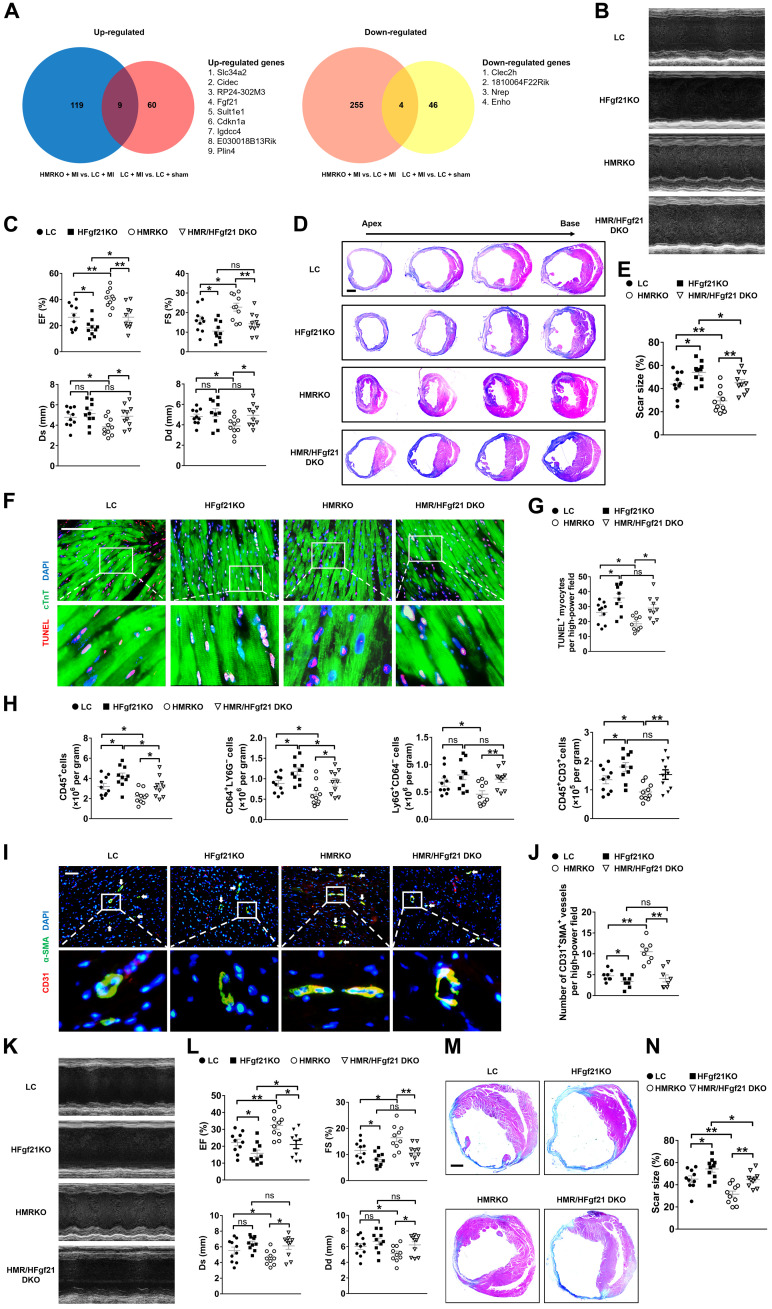
FGF21 mediates the protection of hepatocyte *MR* deficiency on MI. (**A**) Venn diagram presenting the overlap of up-regulated or down-regulated genes between HMRKO + MI versus LC + MI and LC + MI versus LC + sham in mouse livers 1 day after MI. Gene expression was measured by RNA-seq. Up-regulated genes with log_2_fold change > 2 and down-regulated genes with log_2_fold change < −2 are shown. *n* = 3. (**B**) Representative images of echocardiography in mice 1 week after MI. HMR/HFgf21 DKO indicates hepatocyte *MR* and *Fgf21* double knockout. (**C**) Quantifications of EF, FS, Ds, and Dd based on echocardiography. *n* = 10. (**D**) Representative Masson’s trichrome staining of mouse cardiac sections 1 week after MI. Scale bar, 1 mm. (**E**) Quantification of scar size in hearts exemplified in (D). *n* = 10. (**F**) Representative TUNEL staining and cTnT immunofluorescence staining in mouse cardiac sections 3 days after MI. Scale bar, 100 μm. (**G**) Quantification of TUNEL-positive myocytes. *n* = 10. (**H**) Quantifications of flow cytometry analysis of CD45^+^ cells, CD64^+^Ly6G^−^ macrophages, Ly6G^+^CD64^−^ granulocytes, and CD45^+^CD3^+^ lymphocytes in mouse heart samples 3 days after MI. *n* = 10. (**I**) Representative immunofluorescence staining of α-SMA and CD31 in peri-infarct regions of mouse heart samples 7 days after MI. White arrows indicate vascularization. Scale bar, 100 μm. (**J**) Quantification of neovascularization in the peri-infarct regions. *n* = 8. (**K**) Representative images of echocardiography in mice 8 weeks after MI. (**L**) Quantification of EF, FS, Ds, and Dd based on echocardiography. *n* = 10. (**M**) Representative Masson’s trichrome staining of mouse cardiac sections 8 weeks after MI. Scale bar, 5 mm. (**N**) Quantification of scar size in hearts exemplified in (M). *n* = 10. Data are represented as means ± SEM. *P < 0.05, **P < 0.01, and ***P < 0.001.

We next asked what downstream mechanisms mediated the protection of HMRKO on MI. MR in the liver decreased early after MI, and HMRKO protected the heart from MI (Figs. 1 and 2 and fig. S1B), indicating that the decrease of hepatic *MR* after MI was a protective mechanism and that HMRKO might protect the heart from MI by regulation of similar hepatic genes affected by MI with same directions. On the basis of this rationale, we performed RNA-seq in livers from both LC and HMRKO mice 1 day after MI or sham operation (fig. S3A), compared differentially expressed genes between LC + MI versus LC + sham and HMRKO + MI versus LC + MI using Venn diagram, and identified nine up-regulated genes and four down-regulated genes (Fig. 3A). The up-regulated *Fgf21* caught our close attention because, among the 13 genes, it was the only one that encoded a secretory protein, which made it possible for potential communications between the liver and the heart. Results of enzyme-linked immunosorbent assay (ELISA) and qRT-PCR demonstrated that the levels of FGF21 in plasma and livers rose rapidly after MI and peaked at day 1, coincidental with the trough time point of *MR* expression in livers (figs. S1B and S3, B and C). In addition, markedly higher levels of FGF21 were observed in HMRKO than LC mice after MI (fig. S3, B and C).

FGF21 has been previously demonstrated to exert protective effects against cardiovascular diseases (*28*). We hypothesized that hepatocyte *MR* deficiency worked through FGF21 to alleviate cardiac damages after MI. Hepatocyte *MR* and *Fgf21* double-knockout (HMR/HFgf21 DKO) mice were generated to test this hypothesis (fig. S3D). Results of ELISA showed nearly nondetectable levels of FGF21 in plasma from HFgf21KO mice and HMR/HFgf21 DKO mice compared to LC mice 1 and 8 weeks after MI (fig. S3E), indicating that the liver was the major source of circulating FGF21 after MI. Echocardiography of HMR/HFgf21KO DKO mice displayed significantly lower EF and FS, as well as significantly higher Ds and Dd than that of HMRKO mice 1 week after MI (Fig. 3, B and C). Masson’s trichrome staining of cardiac sections of HMR/HFgf21KO DKO mice showed larger infarct scar size than that of HMRKO mice at this time point (Fig. 3, D and E). TUNEL staining showed a significant elevation of apoptotic cardiomyocytes in the border region of hearts from HMR/HFgf21 DKO mice compared to HMRKO mice 3 days after MI (Fig. 3, F and G). Flow cytometry results revealed that MI-induced infiltration of CD45^+^ cells, CD64^+^Ly6G^−^ macrophages, Ly6G^+^CD64^−^ granulocytes, and CD45^+^CD3^+^ lymphocytes was considerably increased in hearts from HMR/HFgf21 DKO mice compared to HMRKO mice (Fig. 3H). Accordingly, qRT-PCR demonstrated that the expression of proinflammatory cytokines was markedly higher in the hearts of HMR/HFgf21 DKO mice than HMRKO mice after MI (fig. S3F). The number of blood vessels in HMR/HFgf21 DKO mouse hearts was decreased compared to that of HMRKO mice 7 days after MI (Fig. 3, I and J, and fig. S3, G and H). Similarly, cardiac function was markedly decreased, and infarct scar size was significantly increased in HMR/HFgf21 DKO mice compared to HMRKO mice at 8 weeks after MI (Fig. 3, K to N). The fibrosis area and cardiomyocyte size in the noninfarct region of HMR/HFgf21 DKO mouse hearts were notably larger than those of HMRKO mice 8 weeks after MI (fig. S3, I and J). HMR/HFgf21 DKO mice also had significantly larger HW/BW, HW/TL, and LW/BW than HMRKO mice (fig. S3K). The expression of HF markers (ANP and BNP) and fibrogenic genes was substantially increased in the noninfarct region of HMR/HFgf21 DKO mouse hearts compared to that of HMRKO mice after MI (fig. S3, L and M). These observations suggested that FGF21 mediated the protection of hepatocyte *MR* deficiency on MI.

Nonetheless, hepatocyte *Fgf21* knockout (HFgf21KO) mice manifested worse cardiac function and remodeling compared to LC mice after MI, further demonstrating the importance of hepatic FGF21 in cardiac protection (Fig. 3 and fig. S3). HMR/HFgf21 DKO mice exhibited better cardiac phenotypes compared to HFgf21KO mice after MI, suggesting that hepatic *MR* deficiency may also work through other mechanisms besides FGF21 to exert cardiac protection (Fig. 3 and fig. S3).

### An MR-NCOR1-HDAC3 complex regulates *Fgf21* expression in hepatocytes

**Fig. 4.**
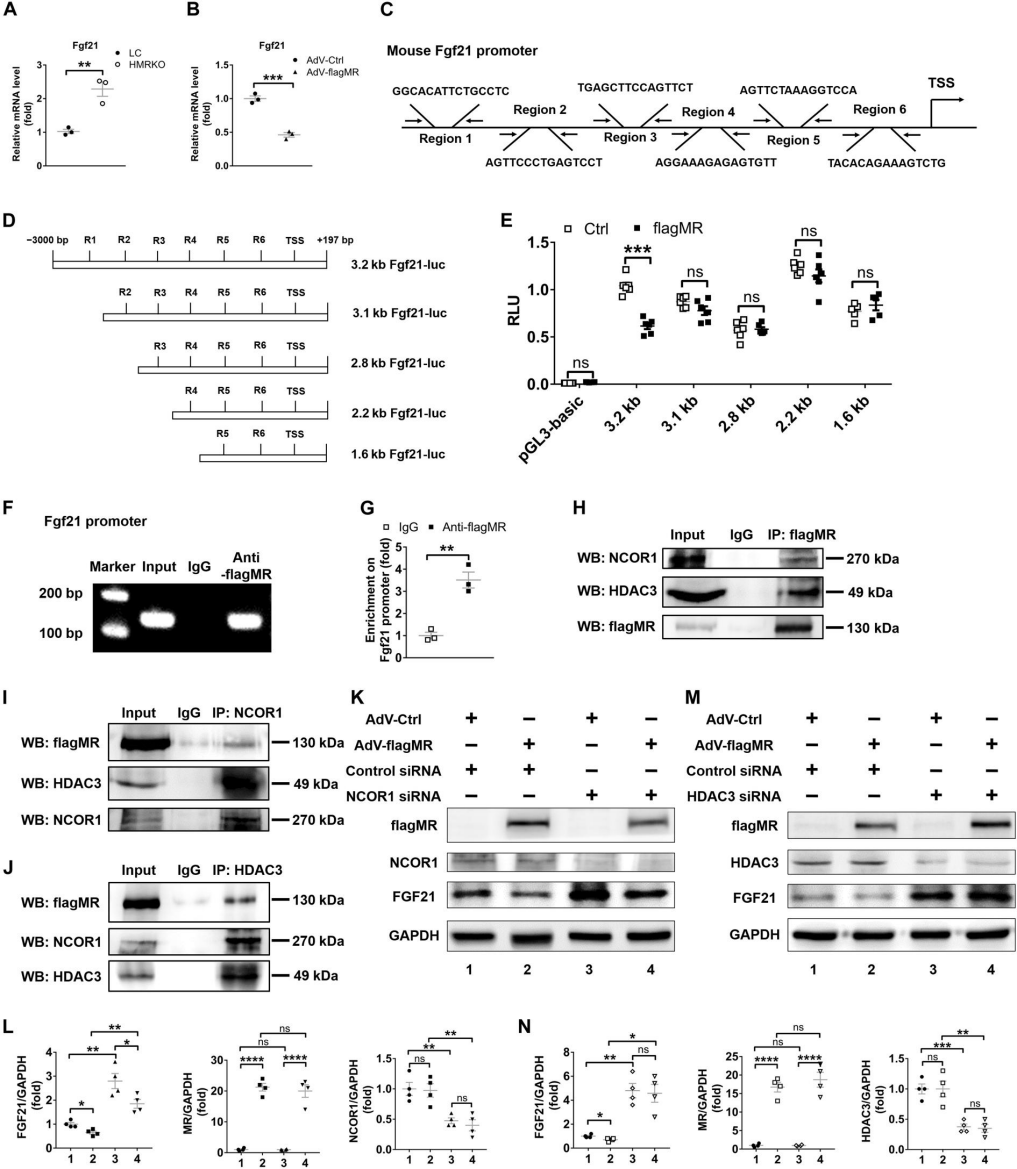
An MR-NCOR1-HDAC3 complex regulates *Fgf21* expression in hepatocytes. (**A**) qRT-PCR analysis of *Fgf21* gene expression in primary hepatocytes from LC and HMRKO mice. (**B**) qRT-PCR analysis of *Fgf21* gene expression in primary hepatocytes infected with control adenovirus (AdV-Ctrl) or flagMR adenovirus (AdV-flagMR) for 48 hours. Multiple of infection = 5. (**C**) Schematic illustration of putative MR binding regions on the promoter of mouse *Fgf21*. TSS, transcription start site. (**D**) Schematic illustration of luciferase reporter constructs containing mouse *Fgf21* promoters (*Fgf21*-luc) with different length. (**E**) Luciferase reporter assays using HEK293FT cells cotransfected with flagMR plasmid or empty vector (Ctrl) and *Fgf21*-luc with different length or control plasmid pGL3-basic. RLU, relative luciferase unit. (**F** and **G**) ChIP analyses showing enrichment of MR on the promoter of *Fgf21* in hepatocytes by regular PCR and gel electrophoresis (F) or qRT-PCR (G). Primers specific to R1 were used for PCR. IgG, immunoglobulin G. (**H** to **J**) Co-immunoprecipitation (IP) analyses of MR, NCOR1, and HDAC3 in primary hepatocytes infected with AdV-flagMR. (**K**) Western blotting (WB) analysis of MR, NCOR1, and FGF21 protein expression in primary hepatocytes infected with AdV-Ctrl or AdV-flagMR for 24 hours and then transfected with control small interfering RNA (siRNA) or *NCOR1* siRNA for 48 hours. GAPDH, glyceraldehyde-3-phosphate dehydrogenase. (**L**) Quantification of Western blotting results exemplified in (K). (**M**) Western blotting analysis of MR, HDAC3, and FGF21 protein expression in primary hepatocytes infected with AdV-Ctrl or AdV-flagMR for 24 hours and then transfected with control siRNA or *HDAC3* siRNA for 48 hours. (**N**) Quantification of Western blotting results exemplified in (M). Data represent three independent experiments. Data are represented as means ± SEM. *P < 0.05, **P < 0.01, ***P < 0.001, and ****P < 0.0001.

Deficiency of hepatocyte *MR* promoted expression of *Fgf21* in mouse livers (fig. S3, B and C) and in primary hepatocytes (Fig. 4A). Conversely, overexpression of MR using flagMR adenovirus (Adv-flagMR) decreased *Fgf21* expression in primary hepatocytes (Fig. 4B). These data indicated a repressive regulation of *Fgf21* by MR in hepatocytes.

Six putative *MR* response elements (MREs) were identified in the promoter region of mouse *Fgf21* (referred as regions 1 to 6, or R1 to R6) (Fig. 4C). Truncated mouse *Fgf21* promoter plasmids were constructed to perform luciferase assays in human embryonic kidney (HEK) 293FT cells (Fig. 4D). The results demonstrated that *MR* suppressed the transcriptional activity of *Fgf21* promoter with full length (Fig. 4E). However, the suppression disappeared when R1 was absent, implying that R1 was required and functional MREs were located in R1 (Fig. 4E). Subsequently, the results of chromatin immunoprecipitation (ChIP) assays using primary hepatocytes infected with Adv-flagMR showed direct binding of *MR* to the *Fgf21* promoter in R1, but not R2 to R6, further supporting the functionality of MREs in R1 (Fig. 4, F and G, and fig. S4A).

We went further to dissect the transcriptional regulation complex involved in the repression of *Fgf21* by MR. Knockdown or inhibition of histone deacetylase 3 (*HDAC3*) promotes expression of FGF21 and attenuates transcriptional activity of MR. Nuclear receptor corepressor 1 (NCOR1) often recruits HDACs to execute its repressive function on gene expression. These results suggest that MR may form a complex with NCOR1 and HDAC3 to exert its inhibitory function on *Fgf21*. Co-immunoprecipitation (Co-IP) analyses revealed interactions among MR, NCOR1, and HDAC3 in primary hepatocytes (Fig. 4, H to J). However, we did not detect interactions between MR and other HDACs, including HDAC1, HDAC5, HDAC6, and HDAC9 (fig. S4B). Next, we tested the functionality of the MR-NCOR1-HDAC3 complex in regulating *Fgf21* expression in hepatocytes. Overexpression of *MR* using Adv-flagMR reduced the expression level of *Fgf21* in primary hepatocytes, and such reduction was reversed by knockdown of either *NCOR1* or *HDAC3* using small interfering RNA (siRNA) (Fig. 4, K to N, and fig. S4, C and D). As a result, hepatocytes with *MR* overexpression and knockdown of *NCOR1* or *HDAC3* had substantially higher expression of *Fgf21* than those with *MR* overexpression alone (Fig. 4, K to N, and fig. S4, C and D). Knockdown of *NCOR1* or *HDAC3* alone increased the expression of *Fgf21* in hepatocytes (Fig. 4, K to N, and fig. S4, C and D). Collectively, these results showed that MR inhibited *Fgf21* expression through interactions with NCOR1 and HDAC3 in hepatocytes.

### Spironolactone ameliorates adverse cardiac remodeling after MI through hepatic FGF21

**Fig. 5.**
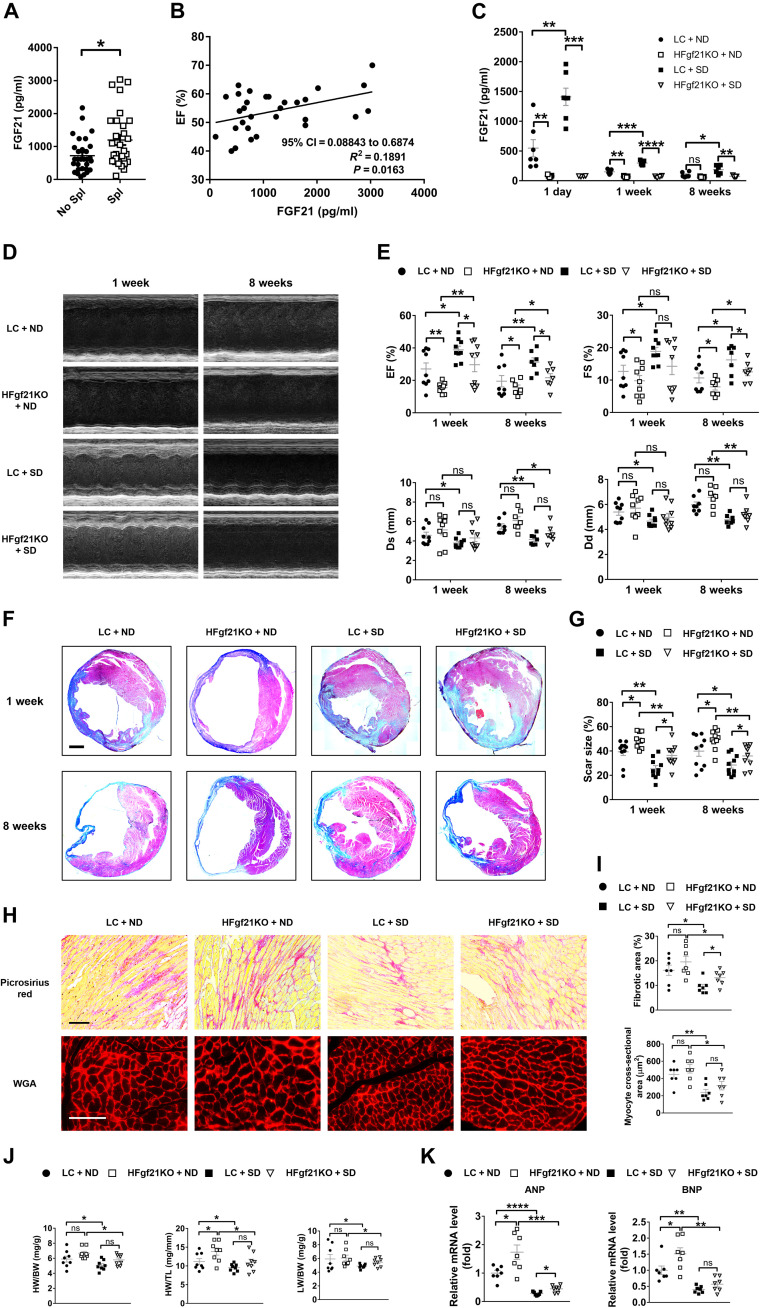
Spironolactone ameliorates adverse cardiac remodeling through hepatic FGF21. (**A**) Plasma levels of FGF21 tested by ELISA in patients with HP treated without or with spironolactone (Spl). *n* = 30. (**B**) Correlation between plasma FGF21 and EF in patients with HP treated with Spl. Pearson correlation test was used. *n* = 30. (**C**) Plasma levels of FGF21 tested by ELISA in mice at different time points after MI. ND, normal diet; SD, spironolactone diet. *n* = 5 to 7. (**D**) Representative images of echocardiography in mice 1 or 8 weeks after MI. (**E**) Quantifications of EF, FS, Ds, and Dd based on echocardiography. *n* = 7 to 9. (**F**) Representative Masson’s trichrome staining of mouse cardiac sections 1 or 8 weeks after MI. Scale bar, 5 mm. (**G**) Quantification of scar size in hearts exemplified in (F). *n* = 10. (**H**) Representative Picrosirius red staining and WGA staining in noninfarct regions of mouse heart samples 8 weeks after MI. Scale bar, 100 μm. (**I**) Quantitative analyses of fibrotic area and cardiomyocyte size. *n* = 7. (**J**) HW/BW, HW/TL, and LW/BW of mice 8 weeks after MI. *n* = 8. (**K**) qRT-PCR analysis of *ANP* and *BNP* gene expression in mouse heart samples 8 weeks after MI. *n* = 7. Data are represented as means ± SEM. *P < 0.05, **P < 0.01, ***P < 0.001, and ****P < 0.0001.

To explore the importance of the MR/FGF21 axis in a more clinically relevant setting, we investigated whether spironolactone, an MR antagonist commonly used to treat HF (*29*, *30*), worked through FGF21 to exert its cardioprotective effects. We first conducted a retrospective cohort study to include 60 patients with HF due to MI. Half of the patients were treated with spironolactone (Spl group; *n* = 30) and the other half were not (no Spl group; *n* = 30). Baseline characteristics of the study population are summarized in table S1. The results of ELISA showed that the plasma FGF21 level of the Spl group was markedly higher than that of the no Spl group 1 month after the initiation of treatments with or without spironolactone (Fig. 5A). Patients in the Spl group demonstrated significantly better cardiac function than those in the no Spl group at this time point (fig. S5A). Plasma FGF21 levels were positively correlated with the EF of patients in the Spl group (Fig. 5B).

We further conducted a retrospective study that included 30 patients with acute MI. Ten of the patients were treated with spironolactone (Spl group; *n* = 10) and 20 patients were not (no Spl group; *n* = 20). Baseline characteristics of the study population are summarized in table S2. The result of ELISA showed that the plasma FGF21 level of the Spl group was markedly higher than that of the no Spl group 3 days after treatments (fig. S5B), consistent with the result obtained from the HF cohort. These clinical data demonstrated that spironolactone promoted FGF21 secretion and improved cardiac function.

We then resorted HFgf21KO mice to further test the importance of hepatic FGF21 in mediating the protective effects of spironolactone on cardiac dysfunction and adverse cardiac remodeling after MI. LC and HFgf21KO mice were fed with spironolactone diet (SD) or normal diet (ND) and then subjected to MI. The results of ELISA illustrated that SD significantly increased FGF21 in the plasma of LC but not HFgf21KO mice (Fig. 5C). HFgf21KO substantially attenuated the effects of SD in improving cardiac function and reducing infarct scar size, as assessed by echocardiography and Masson’s trichrome staining, respectively, in mice fed with SD 1 and 8 weeks after MI (Fig. 5, D to G). Consistently, HFgf21KO diminished the effects of SD in reducing cardiac fibrosis, inhibiting cardiac hypertrophy, and attenuating HF in mice (Fig. 5, H to K). However, we also observed that SD improved cardiac function and remodeling in HFgf21KO mice after MI, suggesting that HFgf21KO did not completely inhibit the effects of spironolactone (Fig. 5, D to K). These data further demonstrated that spironolactone ameliorated MI-induced cardiac dysfunction and adverse cardiac remodeling partially through hepatic FGF21.

### Hepatic IL-6 signaling suppresses *MR* expression and is crucial for cardioprotection after MI in mice

**Fig. 6.**
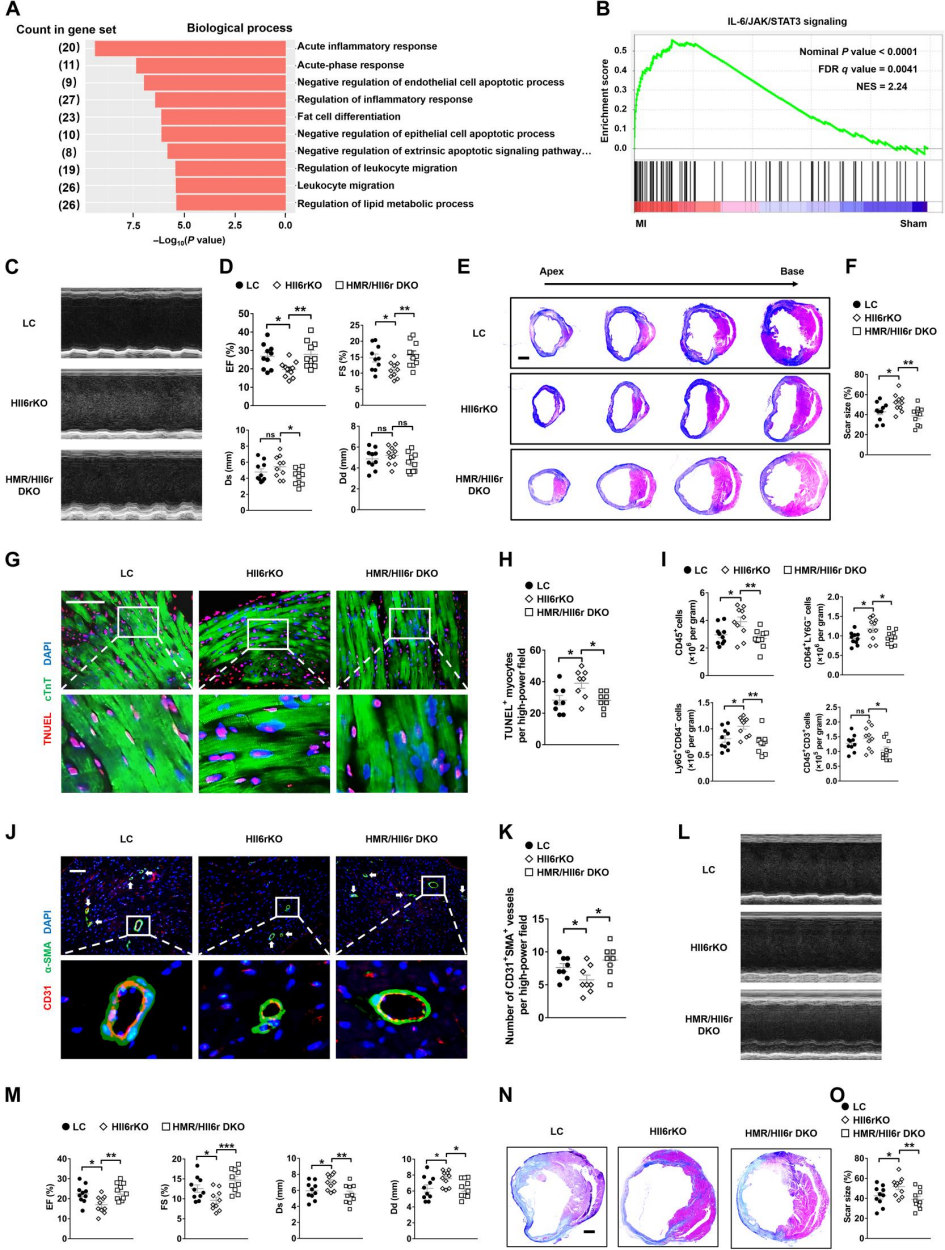
Hepatic *Il6r* suppresses MR expression and exerts cardioprotection after MI in mice. (**A**) Enriched biological processes based on RNA-seq results in livers 12 hours after MI versus sham operation. *n* = 3. (**B**) Enrichment of IL-6/JAK/STAT3 signaling genes based on RNA-seq results in livers 12 hours after MI versus sham operation. *n* = 3. FDR, false discovery rate; NES, normalized enrichment score. (**C**) Representative images of echocardiography in mice 1 week after MI. HMR/HIl6r DKO indicates hepatocyte *MR* and *Il6* receptor double knockout. (**D**) Quantifications of EF, FS, Ds, and Dd based on echocardiography. *n* = 10. (**E**) Representative Masson’s trichrome staining of mouse cardiac sections 1 week after MI. Scale bar, 1 mm. (**F**) Quantification of scar size in hearts exemplified in (E). *n* = 10. (**G**) Representative TUNEL staining and cTnT immunofluorescence staining in mouse cardiac sections 3 days after MI. Scale bar, 200 μm. (**H**) Quantification of TUNEL-positive myocytes. *n* = 8. (**I**) Quantifications of flow cytometry analysis of CD45^+^ cells, CD64^+^Ly6G^−^ macrophages, Ly6G^+^CD64^−^ granulocytes, and CD45^+^CD3^+^ lymphocytes in mouse heart samples 3 days after MI. *n* = 10. (**J**) Representative immunofluorescence staining of α-SMA and CD31 in peri-infarct regions of mouse heart samples 7 days after MI. White arrows indicate vascularization. Scale bar, 100 μm. (**K**) Quantification of neovascularization in peri-infarct regions. *n* = 8. (**L**) Representative images of echocardiography in mice 8 weeks after MI. (**M**) Quantification of EF, FS, Ds, and Dd based on echocardiography. *n* = 10. (**N**) Representative Masson’s trichrome staining of mouse cardiac sections 8 weeks after MI. Scale bar, 1 mm. (**O**) Quantification of scar size in hearts exemplified in (N). *n* = 10. Data are represented as means ± SEM. *P < 0.05, **P < 0.01, and ***P<0.001.

So far, we demonstrated that MR was a critical player in the liver responding to MI and regulating cardiac remodeling through the downstream FGF21. Then, what was the upstream mechanism that controlled the MR/FGF21 signaling after MI? To address this question, we performed RNA-seq of livers from C57 mice at early time point (12 hours) after MI or sham operation (fig. S6A). Acute inflammatory response stood out as the most enriched biological process in the liver after MI (Fig. 6A). Gene set enrichment analysis revealed an enrichment of inflammation-related pathways, particularly the IL-6/Janus kinase (JAK)/signal transducer and activator of transcription 3 (STAT3) signaling (Fig. 6B). Consistently, phosphorylated STAT3 (p-STAT3) was markedly increased in livers at early stage after MI (fig. S6, B and C). Results of ELISA showed that plasma concentration of IL-6 considerably increased at the early stage after MI (fig. S6D). Furthermore, the expression of MR was significantly suppressed while that of *Fgf21* was markedly induced in primary hepatocytes within 2 hours of treatment by IL-6 (fig. S6E).

We therefore hypothesized that the IL-6/STAT3 signaling pathway regulated the MR/FGF21 axis in the liver to exert cardiac protection. Hepatocyte *Il6* receptor knockout (HIl6rKO) mice and hepatocyte *MR/Il6* receptor double knockout (HMR/HIl6r DKO) mice were generated to test the hypothesis. Echocardiography and Masson’s trichrome staining demonstrated that HIl6rKO mice had markedly worse cardiac function and larger infarct scar size than LC mice at 1 week after MI (Fig. 6, C to F). Consistently, HIl6rKO mice showed more apoptotic cardiomyocytes, more leukocyte infiltration, and higher expression of proinflammatory cytokines compared to LC mice 3 days after MI (Fig. 6, G to I, and fig. S6F). The number of blood vessels was also significantly reduced in hearts of HIl6rKO mice compared to that of LC mice 1 week after MI (Fig. 6, J and K, and fig. S6, G and H). Similarly, cardiac function was markedly decreased and infarct scar size was significantly increased in HIl6rKO mice compared to LC mice at 8 weeks after MI (Fig. 6, L to O). Moreover, HIl6rKO mice manifested increased cardiac fibrosis and exacerbated cardiac hypertrophy and HF 8 weeks after MI (fig. S6, I to M). These results showed that IL-6 signaling in the liver was indispensable for cardioprotection after MI in mice.

HMR/HIl6r DKO mice manifested significantly better cardiac function and improved remodeling compared to HIl6rKO mice after MI (Fig. 6, C to O, and fig. S6, F to M), suggesting that IL-6 signaling worked through MR in the liver. Results of qRT-PCR showed that HIl6rKO increased the expression of *MR* and decreased that of *Fgf21* in the liver after MI (fig. S6N). HMR/HIl6r DKO reversed the suppressive effects of HIl6rKO on FGF21, as revealed by both qRT-PCR and ELISA (fig. S6, N and O). These data together suggested that hepatic IL-6 signaling regulated MR/FGF21 axis and mediated the heart-liver communication after MI.

### STAT3 signaling in hepatocytes suppresses MR expression and improves cardioprotection after MI in mice

**Fig. 7.**
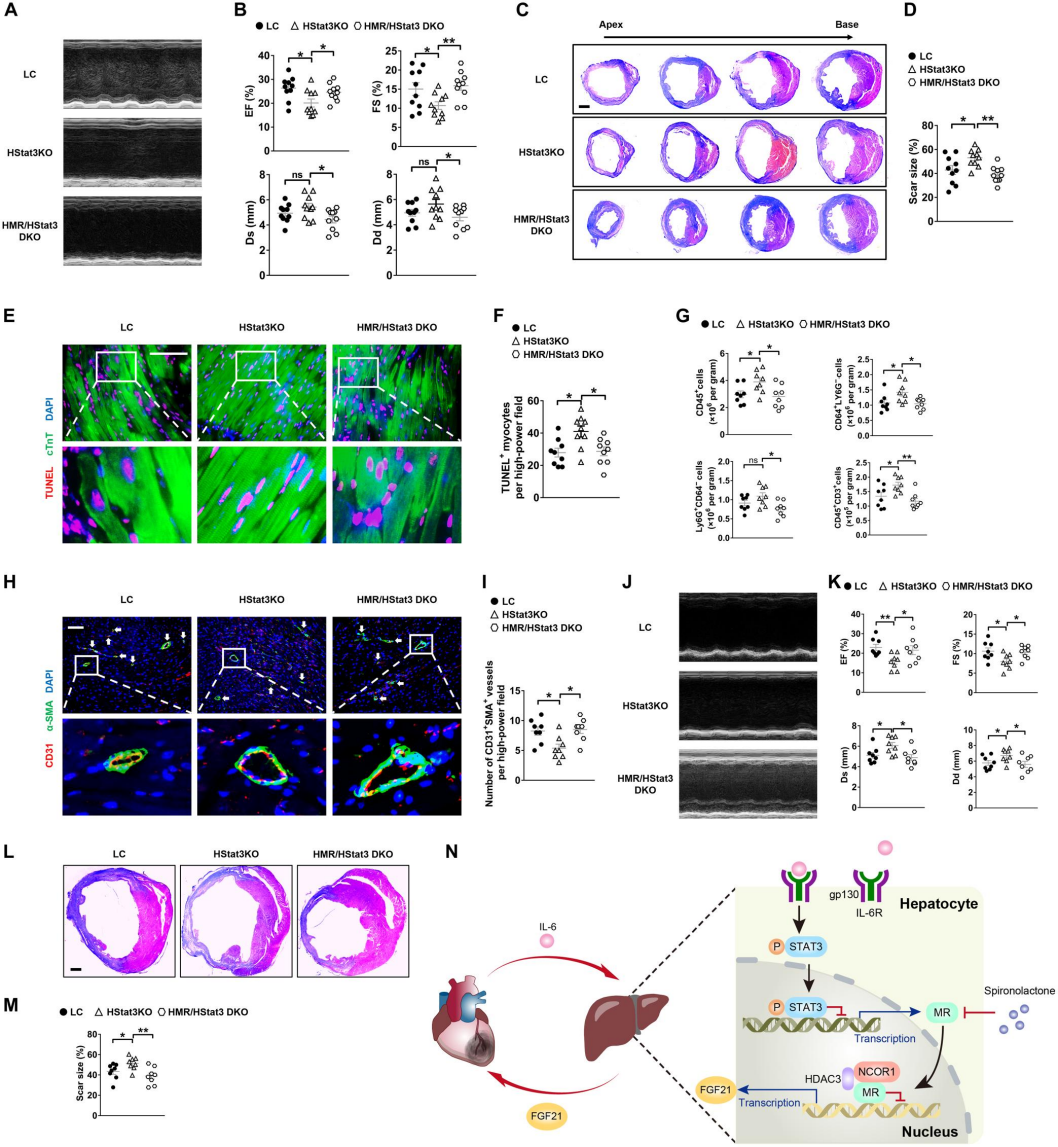
STAT3 signaling in hepatocytes suppresses *MR* expression and improves cardioprotection after MI in mice. (**A**) Representative images of echocardiography in mice 1 week after MI. HMR/HStat3 DKO indicates hepatocyte *MR* and *Stat3* double knockout. (**B**) Quantifications of EF, FS, Ds, and Dd based on echocardiography. *n* = 10. (**C**) Representative Masson’s trichrome staining of mouse cardiac sections 1 week after MI. Scale bar, 1 mm. (**D**) Quantification of scar size in hearts exemplified in (C). *n* = 10. (**E**) Representative TUNEL staining and cTnT immunofluorescence staining in mouse cardiac sections 3 days after MI. Scale bar, 200 μm. (**F**) Quantification of TUNEL-positive myocytes. *n* = 9. (**G**) Quantifications of flow cytometry analysis of CD45^+^ cells, CD64^+^Ly6G^−^ macrophages, Ly6G^+^CD64^−^ granulocytes, and CD45^+^CD3^+^ lymphocytes in mouse heart samples 3 days after MI. *n* = 8. (**H**) Representative immunofluorescence staining of α-SMA and CD31 in peri-infarct regions of mouse heart samples 7 days after MI. White arrows indicate vascularization. Scale bar, 100 μm. (**I**) Quantification of neovascularization in peri-infarct regions. *n* = 8. (**J**) Representative images of echocardiography in mice 8 weeks after MI. (**K**) Quantification of EF, FS, Ds, and Dd based on echocardiography. *n* = 8. (**L**) Representative Masson’s trichrome staining of mouse cardiac sections 8 weeks after MI. Scale bar, 1 mm. (**M**) Quantification of scar size in hearts exemplified in (L). *n* = 8. (**N**) Working model. Data are represented as means ± SEM. *P < 0.05 and **P < 0.01.

To further investigate the role of IL-6/STAT3 signaling pathway in regulation of hepatic MR/FGF21 axis after MI, we generated hepatocyte *Stat3* knockout (HStat3KO) mice and hepatocyte *MR/Stat3* double-knockout (HMR/HStat3 DKO) mice. EF and FS were significantly decreased and infarct scar size was markedly increased in HStat3KO mice compared to LC mice at 1 week after MI (Fig. 7, A to D). Dual deficiency of *MR* and *Stat3* in hepatocytes alleviated the effects of HStat3KO, and consequently, HMR/HStat3 DKO mice manifested significantly improved cardiac function and decreased infarct scar size compared to HStat3KO mice (Fig. 7, A to D). Consistently, HStat3KO promoted apoptosis of cardiomyocytes and infiltration of leukocytes, induced expression of proinflammatory cytokines in hearts 3 days after MI, and reduced the number of blood vessels in hearts 1 week after MI, all of which were attenuated in HMR/HStat3 DKO mice (Fig. 7, E to I, and fig. S7, A to C). Similarly, the detrimental impacts of HStat3KO on cardiac function and remodeling were substantially blunted by HMR/HStat3 DKO in mice 8 weeks after MI (Fig. 7, J to M, and fig. S7, D to H).

Results of qRT-PCR showed that HStat3KO increased the expression of *MR* and decreased that of *Fgf21* in the liver after MI (fig. S7I). The results of ELISA revealed that HMR/HStat3 DKO reversed the suppressive effect of HStat3KO on FGF21 (fig. S7J). Together, these data suggested that hepatic STAT3 also regulated MR/FGF21 axis and mediated the heart-liver communication after MI.

Last, we addressed whether STAT3 directly regulated *MR* in hepatocytes. Hepatocyte *Stat3* deletion increased *MR* gene expression in livers after MI (fig. S7I). Consistently, hepatocyte *Stat3* deletion increased MR protein level in primary hepatocytes (fig. S7, K and L). We identified a putative STAT3 response element in the promoter of mouse *MR* (fig. S7M). ChIP assay revealed direct binding of STAT3 to the *MR* promoter (fig. S7, N and O). Luciferase assays demonstrated that STAT3 suppressed the transcriptional activity of *MR* promoter (fig. S7P). These data demonstrated a direct transcriptional repression on *MR* by STAT3 in hepatocytes.

In summary, we uncover a heart-liver cross-talk mechanism after MI. Inflammatory factor IL-6 triggers liver response to MI at the early stage by activating IL-6/STAT3 signaling in the liver. The IL-6/STAT3 signaling suppresses the expression of *MR*, which, in turn, negatively regulates FGF21 expression through forming a complex with NCOR1 and HDAC3 in hepatocytes. The decrease of MR or blockade of MR by spironolactone promotes the expression of FGF21, which exerts cardioprotective roles in an endocrine manner (Fig. 7N).

## DISCUSSION

The cross-talk between the heart and the liver in response to cardiac injury has remained poorly understood. In the present study, we demonstrated that MR decreased markedly after MI and that both hepatic *MR* deficiency and spironolactone improved cardiac repair through regulation of hepatic FGF21, presenting a mechanism underlying the protective effects of the liver on the heart. We also revealed that acute activation of IL-6/STAT3 signal was responsible for the suppression of MR in the liver and that both hepatic *Il6r* deficiency and hepatic *STAT3* deficiency exacerbated MI-induced cardiac injury through regulation of hepatic MR/FGF21, uncovering an upstream path for the liver to receive signals from the heart and to exert cardioprotection. Therefore, an IL-6/STAT3/MR/FGF21 signaling axis mediated the cross-talk between the heart and the liver after cardiac injury.

First, we have identified a hepatic MR/FGF21 axis that mediates the liver-to-heart protection against MI. Although previous reports have shown that the liver may play a protective role against MI (*9*, *10*), the regulatory mechanism and key control points have remained unclear. Our results revealed that MR was among the substantially suppressed transcription factors and one of the three substantially altered nuclear receptors in the liver after MI. Deficiency of *MR* in cardiomyocytes, myeloid cells, T cells, and vascular smooth muscle cells has been shown to inhibit adverse cardiac remodeling (*26*, *31*–*33*). We demonstrated that hepatic *MR* deficiency was protective against cardiac injury and adverse remodeling after MI. These results suggested MR as a regulatory nexus to effectively convey the liver-to-heart protection against MI. Our results further illustrated that MR, in turn, regulated FGF21, a secretory molecule that has been shown to be beneficial for cardiovascular repair (*28*), transcriptionally in hepatocytes, and that hepatic *MR* deficiency worked through FGF21 to exert its cardioprotective function. Previous studies have implied that the expression of *Fgf21* in hepatocytes can be regulated by other transcription factors such as PPARα (*34*), X-box binding protein 1 (*35*), and retinoic acid receptor β (*36*) under the setting of metabolic regulation. However, it has not been reported which transcription factors regulate the hepatic expression of FGF21 in cardiovascular disease models such as MI. We identified *Fgf21* as a target gene of MR and further delineated the regulatory machinery of an MR-NCOR1-HDAC3 complex that negatively regulated FGF21 in hepatocytes. These data have demonstrated that MR acts as a critical regulator to control the expression of FGF21 in collaboration with NCOR1and HDAC3 in hepatocytes, allowing the liver-to-heart communication during MI.

Second, we have found the upstream IL-6/STAT3 signaling pathway that conveys the heart-to-liver signal to down-regulate MR in the liver and that subsequently exerts cardioprotective effects. We described an early activation of acute inflammatory response, particularly the IL-6/STAT3 pathway, in the liver after MI. Hepatic *Il6r* deficiency and *STAT3* deficiency both exacerbated cardiac injury and adverse remodeling through their regulation on *MR*. These results completed the heart-to-liver leg of the heart-liver cross-talk after MI, highlighting the importance of acute inflammation. Tocilizumab (TCZ), an anti–IL-6 receptor antibody, has been widely used in the treatment of rheumatoid arthritis (*37*). A previous clinical study has shown that TCZ increases serum lipids such as total cholesterol, low-density lipoprotein cholesterol, and triglycerides in patients with rheumatoid arthritis (*38*), posing potential adverse effects on MI, although high-density lipoprotein cholesterol is also increased in these patients (*38*) and more direct evidence is required to draw any conclusion. We demonstrated that hepatic *Il6r* deficiency worsened MI, suggesting that IL-6 receptor antagonists such as TCZ could have a detrimental effect on cardiac function in the setting of MI by inhibiting hepatic IL-6 signaling. Therefore, reducing the accumulation of IL-6 receptor antagonists in the liver could lower the potential cardiac side effects of these drugs.

It is well known that chronic inflammation is detrimental to various repairing processes, including cardiac repair (*39*, *40*). However, acute inflammation is usually necessary and beneficial for these repairing processes, although its importance is often overlooked (*39*, *40*). For instance, depletion of inflammatory cells such as macrophages and neutrophils during acute phase of MI markedly worsens cardiac dysfunction and increases the mortality of mice (*41*, *42*). An important mechanism being proposed is that these early inflammatory cells are instrumental in removing dead cells and paving the way for the repair (*40*, *42*).

Our results presented another mechanism for the beneficial effects of acute inflammation, sending out signals asking for help from the liver through the acute activation of IL-6/STAT3 pathway. IL-6 is an important inflammatory factor with pleiotropic effects in different tissues and plays divergent roles in cardiovascular diseases (*43*). For example, both chronic administration and genetic deficiency of IL-6 have been reported to worsen atherosclerosis in *ApoE* knockout mice (*44*, *45*). These seemly contradictory results may be explained by the fact that IL-6 has different or even the opposite impacts on a disease depending upon the stage of the disease—in general, beneficial in acute phase and detrimental in chronic phase—and cell type–specific effects (*46*). Global deletion of *IL6* ameliorates cardiac remodeling after MI, maybe reflecting the chronic deleterious impacts of IL-6 on the heart during cardiac repair (*47*). Our results demonstrated that blocking the acute activation of IL-6/STAT3 pathway in the liver by hepatic deficiency of *Il6r* or *STAT3* impaired cardiac repair, illustrating the beneficial effects of acute inflammation and the importance of this hepatic pathway in mediating the heart-liver cross-talk after MI. We identified *MR* as a target gene of STAT3 in hepatocytes, and the IL-6/STAT3 pathway worked through the MR/FGF21 axis in the liver.

Last, our results have suggested that targeting hepatic MR pharmacologically may lead to better therapeutic effectiveness, which is of important clinical significance. The success of the first and second generations of MR antagonists (spironolactone and eplerenone, respectively) for treating cardiovascular diseases has been unequivocally illustrated (*48*). Recent clinical trials have shown that finerenone, a selective nonsteroidal MR antagonist (the third and newest generation), reduces risks of chronic kidney disease progression and cardiovascular events in patients with chronic kidney disease and type 2 diabetes (*49*, *50*), with the expectation to have broader cardiovascular indications (*51*). Understanding cell type– or tissue-specific functions of MR and its antagonists may have important implications for designing new compounds to achieve higher efficacy and fewer side effects. For instance, finerenone is expected to have less side effects, particularly hyperkalemia, given its at least three times lower distribution in the kidney relative to the heart in comparison with eplerenone (*52*).

We showed that *MR* decreased most markedly in the liver after MI among a variety of tissues we assessed, indicating that hepatic MR was critical in response to cardiac injury and that targeting hepatic MR was an effective strategy to improve cardiac repair after MI. Our results further demonstrated that both hepatic *MR* deficiency and spironolactone worked through hepatic FGF21 to exert its protection against MI, indicating the effectiveness of targeting hepatic MR. The liver most likely accumulates much more MR antagonists than the heart and kidney given its much bigger mass and similar or higher distribution rate (*52*, *53*). It is probable that MR antagonists exert their cardiovascular protection through the liver more than the heart, although this warrants more precise comparisons in the future. Higher distribution in the liver may become a general pursuit of future generations of MR antagonists aiming to increase efficacy while aiming to decrease side effects (presumably lower distribution in the kidney). It is conceivable to achieve high benefit-to-harm ratio for treating MI and HF by liver-specific delivery of MR antagonists, monoclonal antibodies against MR, or even *MR*-editing CRISPR-Cas9 system using strategies such as nanotechnology and hepatocyte-specific promoters (*54*, *55*).

In summary, we have depicted a heart-liver cross-talk mechanism that the ischemic heart sends signals to the liver through acute inflammatory IL-6/STAT3 pathway and that the liver responds by down-regulating *MR* to promote FGF21 production and to alleviate ischemic injury. These data have identified hepatic MR as a sensor—responds to acute IL-6/STAT3 signaling after MI—and as a regulator—controls the production of the cardioprotective FGF21. Targeting the heart-liver cross-talk and the mediating IL-6/STAT3/MR/FGF21 signaling axis may provide novel strategies to improve cardiac repair after MI and ultimately the treatment for HF.

## MATERIALS AND METHODS

### Mice

HMRKO (*MR*flox/flox; Alb-Cre) and LC (*MR*flox/flox) mice were generated by crossing *MR*flox/flox mice (*56*) with albumin-Cre (Alb-Cre) recombinase transgenic mice (*57*). *Fgf21*flox/flox mice (*58*) were mated with HMRKO mice to generate the following four experimental groups: LC (*MR*flox/flox/*Fgf21*flox/flox), HFgf21KO (*Fgf21*flox/flox, Alb-Cre), HMRKO, and HMR/HFgf21 DKO (*MR*flox/flox/*Fgf21*flox/flox, Alb-Cre). *Il6r*flox/flox mice (*59*) were bred to HMRKO mice to generate LC (*MR*flox/flox/*Il6r*flox/flox) mice, HIl6rKO (*Il6r*flox/flox, Alb-Cre) mice, and HMR/HIl6r DKO (*MR*flox/flox/*Il6r*flox/flox, Alb-Cre) mice. *Stat3* flox/flox mice (*60*) were bred to HMRKO mice to generate LC (*MR*flox/flox/*Stat3*flox/flox) mice, HStat3KO (*Stat3*flox/flox, Alb-Cre) mice, and HMR/HStat3 DKO (*MR*flox/flox/*Stat3*flox/flox, Alb-Cre) mice. Male C57BL/6 mice were purchased from Vital River Laboratory Animal Technology Co. Ltd. (Beijing, China). All animal studies were approved by the Institutional Review and Ethics Board of Shanghai Ninth People’s Hospital, Shanghai Jiao Tong University School of Medicine.

### Human samples

All subjects with HF provided written informed consent, and the study was approved by the Human Ethics Committee of Shanghai Chest Hospital. The retrospective study cohort of patients with HF was derived from the registry of patients at Shanghai Chest Hospital. Inclusion criteria were patients with HF due to MI, older than 18 years of age, and being seen between 2019 and 2021. Exclusion criteria were (i) patients who had used spironolactone or similar drugs within 6 months; (ii) patients with renal insufficiency with blood creatinine of ≥256 μM; (iii) patients with hyperkalemia with blood potassium of ≥5.5 mM; (iv) patients with hepatic insufficiency; (v) patients with acidosis; (vi) patients with enlarged breasts or menstrual disorders; and (vii) patients with tumors, severe infections, autoimmune diseases, etc. Sixty patients were eventually included in the study. Half of the patients were treated with spironolactone (20 mg, orally, once daily) and the other half were not. Baseline characteristics of the patients are described in table S1. The first date after the first follow-up visit was defined as the date of the study entry. At 1-month follow-up visits, peripheral blood samples of patients were collected in anticoagulation tubes, immediately centrifuged, and stored at −80°C until assay. The cardiac function of the patients was assessed by echocardiography.

All subjects with acute MI provided written informed consent and were recruited with approval by the Human Ethics Committee of Shanghai Ruijin Hospital. Inclusion criteria were patients with acute MI, older than 18 years of age, and being treated with percutaneous coronary intervention. The exclusion criteria were the same as above. The retrospective cohort study included 30 patients with MI. Ten of the patients were treated with spironolactone (20 mg, orally, once daily) and 20 patients were not. The first day after the percutaneous coronary intervention was defined as the date of the study entry. Peripheral blood samples of patients were collected 3 days after treatments with or without spironolactone.

### MI mouse model

MI was induced by permanent ligation of the left anterior descending coronary artery in male mice at 10 to 12 weeks of age as previously described (*61*). Briefly, after mice were anesthetized with 2% isoflurane, their hearts were extruded through a small hole made at the left thoracic fourth intercostal space. The left anterior descending coronary artery was located and ligated at a site approximately 3 mm from lower margin of the left atrial appendage using a 6-0 silk suture. After ligation, the heart was instantly put back into the chest cavity followed by manual evacuation of air and suture of muscle and skin. Sham surgeries underwent the same procedure except the coronary artery ligation.

Spironolactone (Minsheng Pharmaceuticals, Hangzhou, China) was incorporated into rodent chow diet at a concentration of 0.1 mg/g of chow. The estimated dose of spironolactone was 20 mg/kg per day, which was commonly used in rodents with MI (*62*, *63*). Mice were fed with SD or ND starting from 5 days before MI operation until the end of experiments.

### Echocardiography analysis

Transthoracic echocardiography was performed after MI using a Vevo 3100 Ultrasound system (VisualSonics, Toronto, Canada). Mice were anesthetized with 1.5% isoflurane, and the heart rate of mice was preserved at approximately 550 bpm. Two-dimensional parasternal long-axis views of the left ventricle were obtained for guided M-mode measurements of the Dd and Ds. EF and FS were measured based on M-mode images.

### Histology and morphometric analysis

Hearts were fixed with 4% paraformaldehyde for 24 hours and embedded in paraffin. Five-micrometer-thick paraffin sections were stained with Masson’s trichrome (Sigma-Aldrich, USA), 0.1% picrosirius red (Sigma-Aldrich), or wheat germ agglutinin (WGA; W32464, Thermo Fisher Scientific). The area of cardiac fibrosis and the scar size were quantified as the percentage of the positively stained area to the total area using Image-Pro Plus software. Cardiomyocyte size was measured using ImageJ software.

### Immunofluorescence staining

Immunofluorescence staining was performed as previously described (*25*). Briefly, paraffin sections of hearts were deparaffinized and rehydrated. The slides were incubated in blocking buffer at room temperature after antigen retrieval treatment, and then incubated with primary antibodies and fluorochrome-conjugated secondary antibodies sequentially. Last, the slides were counterstained with 4′,6-diamidino-2-phenylindole (DAPI).

To detect apoptosis of cardiomyocytes, cardiac sections were incubated with troponin T-C antibody (cTnT; sc-20025, Santa Cruz Biotechnology Inc., Santa Cruz, CA) followed by fluorochrome-conjugated secondary antibodies. TUNEL staining was performed using an In Situ Cell Death Detection kit (Roche) according to the manufacturer’s instructions. Last, the slides were counterstained with DAPI (D1306, Invitrogen).

### Flow cytometry

Heart tissues were analyzed by flow cytometry as previously described (*64*). Briefly, hearts were digested with digestion solution containing collagenase II (1.5 mg/ml; A004174, Worthington), collagenase IV (1.5 mg/ml; A004186, Worthington), and deoxyribonuclease I (60 U/ml; A3778, AppliChem, Lochem, Darmstadt, Germany) in Hank’s balanced salt solution. Cardiac single-cell suspensions were gained by filtering digested tissues through 70-μm cell strainers (BD Biosciences, San Jose, CA, USA). Single-cell suspensions were blocked with Fc block and then stained with CD45-PE-CY7 (103114, BioLegend), CD11b–fluorescein isothiocyanate (101205, BioLegend), CD64-allophycocyanin (139305, BioLegend), Ly6G-phycoerythrin (127607, BioLegend), and CD3-PE (100205, BioLegend). Samples were analyzed using LSRFortessa (BD Biosciences).

### Enzyme-linked immunosorbent assay

Plasma samples were collected from mice or patients and stored at −80°C. FGF21 concentrations were detected using a mouse-specific ELISA kit (MF2100, R&D Systems, Minneapolis, MN) or human-specific ELISA kit (DF2100, R&D Systems) according to the manufacturer’s instructions. IL6, IL-1β, and TNFα were measured using mouse ELISA kits (M6000B, MLB00C, and MTA00B, R&D Systems).

### Quantitative RT-PCR

Total RNA from tissues or cells was extracted using TRIzol (Thermo Fisher Scientific), and cDNA was synthesized using reverse transcription kits (Takara, Shiga, Japan) according to the manufacturer’s instructions. Quantitative PCR was performed using SYBR Green Mix (Thermo Fisher Scientific) on a LightCycler 480II (Roche). Primer sequences are listed in table S3. The level of target gene expression was normalized to the glyceraldehyde-3-phosphate dehydrogenase (*GAPDH*) gene.

### Western blotting

Total proteins were extracted from tissues or cells with radioimmunoprecipitation assay buffer containing protease inhibitors (Beyotime Biotechnology, Shanghai, China) and quantified using the BCA Protein Assay Kit (Pierce, Rockford, IL). Protein samples were separated by SDS–polyacrylamide gel electrophoresis and transferred to polyvinylidene difluoride membranes (Millipore, Bedford, MA). The membranes were immunoblotted with primary antibodies at 4°C overnight, followed by incubation with horseradish peroxidase–conjugated secondary antibodies (1:1000; Proteintech, Chicago, IL). The protein bands were visualized with an ECL detection kit (Thermo Fisher Scientific) and quantified using ImageJ software. The following primary antibodies were used: MR (1:500; ab41912, Abcam), FGF21 (1:1000; AF3057, R&D Systems), cleaved caspase-3 (1:1000; 9664S, Cell Signaling Technology), NCOR1 (1:500; 5948S, Cell Signaling Technology), anti-FLAG (1:2000; F1804, Sigma-Aldrich), HDAC1 (1:500; 39531, Active Motif), HDAC3 (1:1000; 85057S, Cell Signaling Technology), HDAC5 (1:1000; ab55403, Abcam), HDAC6 (1:500; 7612S, Cell Signaling Technology), HDAC9 (1:500; ab59718, Abcam), p-STAT3 (1:1000; 9145S, Cell Signaling Technology), STAT3 (1:1000; 9139S, Cell Signaling Technology,), α-tubulin (1:5000; T6199, Sigma-Aldrich), and GAPDH (1:5000; 5174S, Cell Signaling Technology).

### RNA-seq analysis

Mouse livers were preserved in RNAlater and sent to WuXi NextCODE (Shanghai, China) for sequencing. After RNA quality check, a sequencing library was created using TruSeq technology. Sequencing was performed on a HiSeq X Ten (Illumina). Differentially expressed genes were identified by over twofold change in gene expression with an adjusted value of *P* < 0.05. For heatmap visualization of differentially expressed genes, data were *z* score normalized. Gene set enrichment analysis (www.gsea-msigdb.org/gsea/index.jsp) was performed to identify biological processes that were differentially enriched in experimental groups. The accession number for the high-throughput RNA-seq in this paper is available in Gene Expression Omnibus dataset (RNA-seq data: GSE193516).

### Cell culture and treatment

Primary hepatocytes were isolated from 8-week-old male mice by in situ collagenase perfusion as previously described (*65*). Briefly, mouse livers were perfused with digestion media containing collagenase IV (A004186, Worthington) for 10 min, followed by purification with 50% Percoll (GE Healthcare, Marlborough, MA). Primary hepatocytes were cultured in Medium 199 (Thermo Fisher Scientific, Carlsbad, CA, USA) with 10% fetal bovine serum (FBS; Biological Industries, Kibbutz Beit-Haemek, Israel) and 1% penicillin-streptomycin (Thermo Fisher Scientific). Hepatocytes were treated with IL-6 (20 ng/ml; I9646, Sigma-Aldrich, St. Louis, MO, USA). A recombinant adenoviral vector expressing flag was constructed to make Adv-flagMR plasmid by Hanbio Company (Shanghai, China). Primary hepatocytes were isolated and cultured for 24 hours before infected with Adv-flagMR or Adv-flag (control). HEK293FT cells were obtained from Cell Bank of Chinese Academy of Sciences and cultured in Dulbecco’s modified Eagle’s medium with 10% FBS and 1% penicillin/streptomycin. Cells were maintained in a 5% CO_2_ incubator at 37°C.

### Plasmids and siRNA

A 3-kb mouse Fgf21 promoter was amplified and cloned into pGL3-basic vector (Promega) to produce the 3-kb Fgf21-luciferase plasmid. Different fragments of Fgf21 promoter were cloned into pGL3-basic vector to yield various truncated Fgf21-luciferase plasmids. Similarly, a 2-kb mouse MR promoter was cloned into the pGL3-basic vector and produced the 2-kb MR-luciferase plasmid. The sequences of the primers used to amplify these promoters are listed in table S4.

*HDAC3* siRNA, *NCOR1* siRNA, and their respective control were purchased from Santa Cruz Biotechnology Inc. Primary hepatocytes were transfected with siRNA or control using Lipofectamine 2000 (Thermo Fisher Scientific) according to the manufacturer’s instructions.

### Luciferase reporter assay

Luciferase assays were performed in HEK293FT cells, which were cotransfected with an empty pGL3-basic vector or the promoter constructs together with Renila plasmids using Lipofectamine 2000 (Thermo Fisher Scientific). Cells were harvested and measured with a Dual-Luciferase Reporter Assay kit (Promega, Fitchburg, WI, USA; catalog no. E1910) 48 hours after transfection.

### Co-IP and ChIP

Co-IP experiments were performed in primary hepatocytes from wild-type mice as described previously (*24*). Briefly, cells were lysed in IP lysis buffer containing protease inhibitor cocktail (P8340, Sigma-Aldrich). The cell lysates were mixed with primary antibody or normal IgG (sc-2027 or sc-2025, Santa Cruz Biotechnology), rotated overnight at 4°C, and followed by incubation with protein A/G beads (sc-2003; Santa Cruz Biotechnology Inc., Santa Cruz, CA) at 4°C for 4 hours. The supernatant of the mixture after centrifugation was used for Western blotting analysis.

For ChIP, primary hepatocytes were harvested 48 hours after infection with Adv-flagMR. ChIP experiments were performed using an EZ-ChIP kit (17–371; Millipore, Darmstadt, Germany) according to the manufacturer’s instructions. ChIP products were used for PCR analysis. The sequences of PCR primers are listed in table S5.

### Statistical analysis

All data were expressed as means ± SEM. The analysis of data was performed by Prism (GraphPad Software, La Jolla, CA). Student’s *t* test was used for pairwise comparisons. Multiple comparisons were analyzed by two-way analysis of variance (ANOVA) followed by Bonferroni posttests. Correlation analysis was performed by Pearson correlation test. A value of *P* ≤ 0.05 was considered statistically significant.

## Notes

View/request a protocol for this paper from *Bio-protocol*.
