# Supplementary material for: An IL-6/STAT3/MR/FGF21 axis mediates heart-liver cross-talk after myocardial infarction
Source: Sci Adv. 2023 Apr 5;9(14):eade4110. doi: 10.1126/sciadv.ade4110 (PMC10075967; doi:10.1126/sciadv.ade4110)
Supplement: Supplementary file 1 — Figs. S1 to S7 Tables S1 to S5 [file sciadv.ade4110_sm.pdf]

Science Advances
AAAS

Supplementary Materials for

An IL-6/STAT3/MR/FGF21 axis mediates heart-liver cross-talk after myocardial infarction

Jian-Yong Sun *et al.*

Corresponding author: Xiaoxiang Yan, cardexyanxx@hotmail.com; Ruo-Gu Li, 13564565961@163.com; Sheng-Zhong Duan, duansz2008@hotmail.com

Sci. Adv. **9**, eade4110 (2023)
DOI: 10.1126/sciadv.ade4110

This PDF file includes:

Figs. S1 to S7
Tables S1 to S5

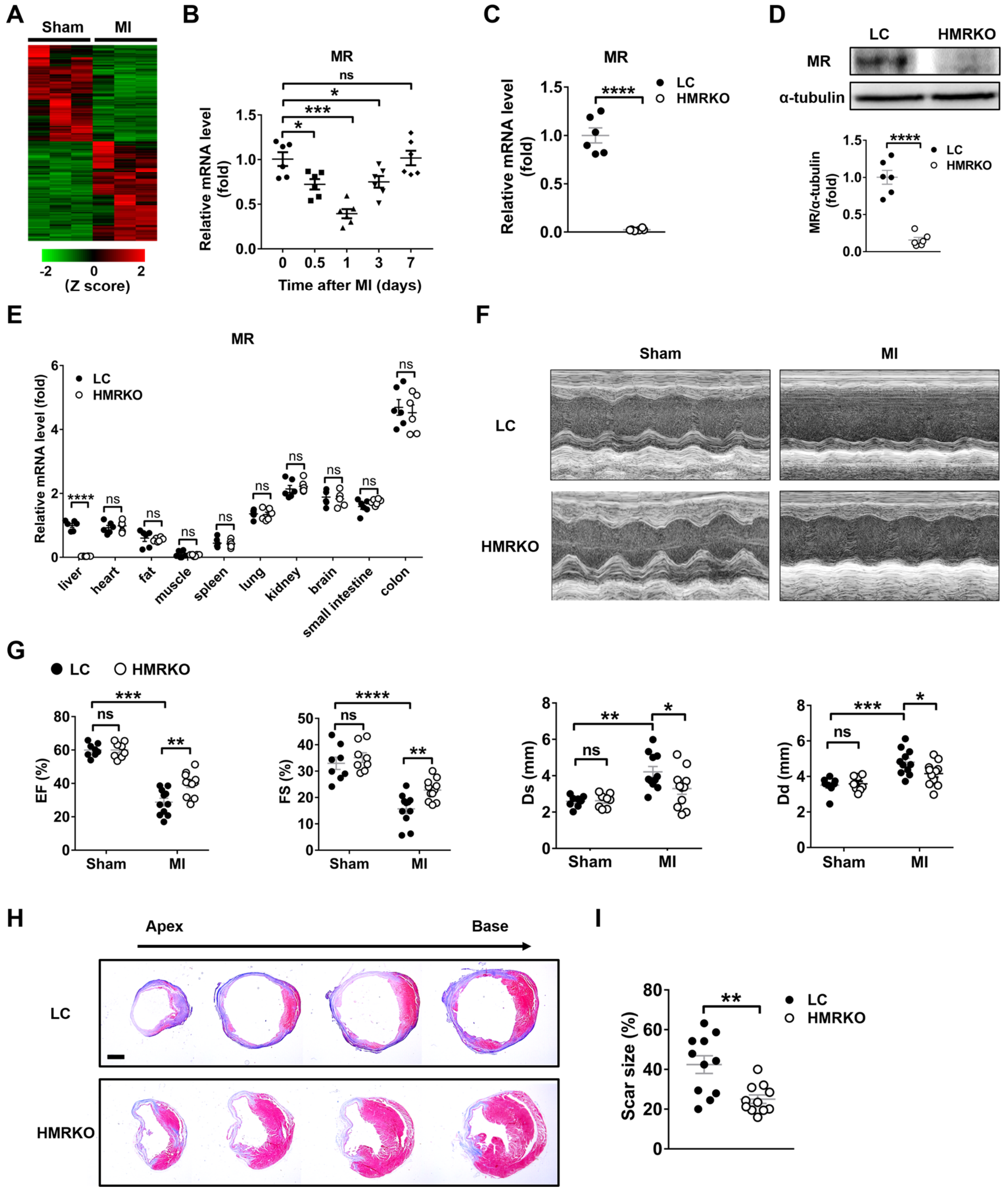

Fig. S1. Deficiency of hepatocyte *MR* improves cardiac function and decreases scar size after MI in mice. (A) Heatmap of RNA-seq results showing differentially expressed genes in livers 1 day after MI versus sham operation. n=3. (B) QRT-PCR analysis of *MR* gene expression in mouse livers at different time points after MI. n=6. (C) QRT-PCR analysis of *MR* gene expression in livers of littermate control (LC) and hepatocyte *MR* knockout (HMRKO) mice. n=6. (D) Western blotting analysis of MR protein expression in mouse livers. n=6. (E) QRT-PCR analysis of *MR* gene expression in different tissues of LC and HMRKO mice. n=6. (F) Representative images of echocardiography in mice 4 weeks after MI. (G) Quantifications of EF, FS, Ds, and Dd based on echocardiography. Sham n=8, MI n=11. (H) Representative Masson's trichrome staining of mouse cardiac sections 4 weeks after MI. Scale bar, 1 mm. (I) Quantification of scar size in hearts exemplified in (H). n=11. Data are represented as mean ± SEM. ns, not significant. *p<0.05, **p<0.01, ***p<0.001, ****p<0.0001.

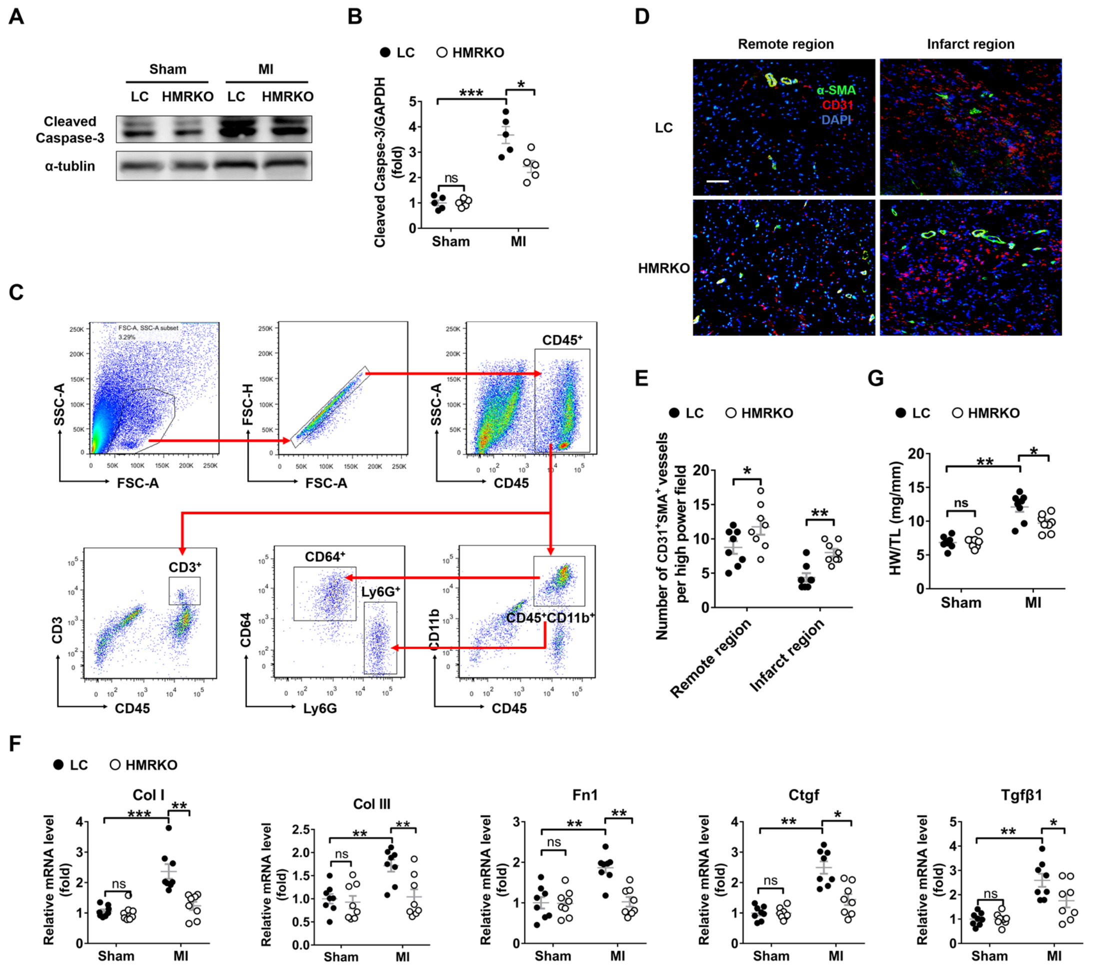

Fig. S2. Deficiency of hepatocyte *MR* inhibits adverse cardiac remodeling after MI in mice. (A) Western blotting analysis of cleaved caspase-3 in mouse hearts 3 days after MI. n=5. (B) Quantification of A. (C) Gating strategy for flow cytometry analysis of heart samples. (D) Representative immunofluorescence staining of α-SMA and CD31 in remote regions and infarct regions of mouse heart samples 7 days after MI. Scale bar, 100 μm. (E) Quantification of neovascularization (α-SMA and CD31 dual-positive area) in remote regions and infarct regions. n=8. (F) QRT-PCR analysis of fibrogenic genes in mouse heart samples 8 weeks after MI or sham operation. n=8. (G) Heart weight to tibia length ratio (HW/TL) of mice 8 weeks after MI or sham operation. n=8. Data are represented as mean ± SEM. ns, not significant. *$p<0.05$, **$p<0.01$.

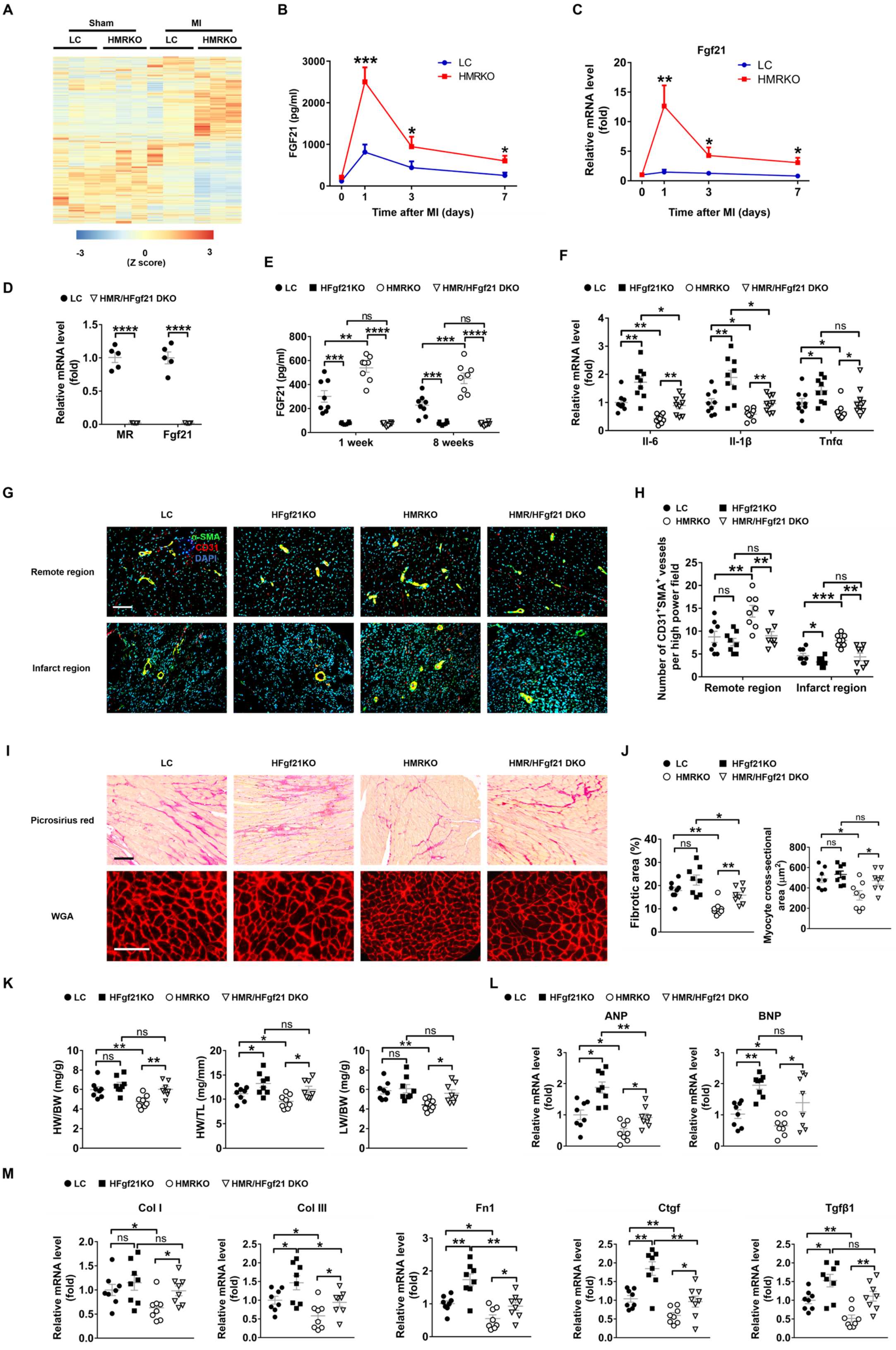

Fig. S3. FGF21 mediates the protection of hepatocyte *MR* deficiency on MI. (A) Heatmap of RNA-seq results showing differentially expressed genes in livers from LC and HMRKO mice 1 day after MI versus sham operation. n=3. (B) Plasma levels of FGF21 tested by ELISA in mice at different time points after MI. n=8. (C) QRT-PCR analysis of *Fgf21* gene expression

in mouse livers at different time points after MI. n=8. (D) QRT-PCR analysis of *MR* and *Fgf21* gene expression in mouse livers. N=5. I Plasma levels of FGF21 tested by ELISA in mice 1 week or 8 weeks after MI. n=8. (F) QRT-PCR analysis of pro-inflammatory cytokines in mouse hearts 3 days after MI, n=9. (G) Representative immunofluorescence staining of α-SMA and CD31 in remote regions and infarct regions of mouse heart samples 7 days after MI. (H) Quantification of neovascularization in remote regions and infarct regions. N=8. (I) Representative Picrosirius red staining and WGA staining of non-infarct regions of mouse heart samples 8 weeks after MI. (J) Quantitative analyses of fibrotic area and cardiomyocyte size. N=8. (K) HW/BW, HW/TL, and LW/BW of mice 8 weeks after MI. n=8. (L) QRT-PCR analysis of *ANP* and *BNP* in heart samples 8 weeks after MI. n=8. (M) QRT-PCR analysis of fibrogenic gene expression in heart samples 8 weeks after MI. n=8. All scale bars, 100 μm. Data are represented as mean ± SEM. Ns, not significant. *p<0.05, **p<0.01, ***p<0.001, ****p<0.0001.

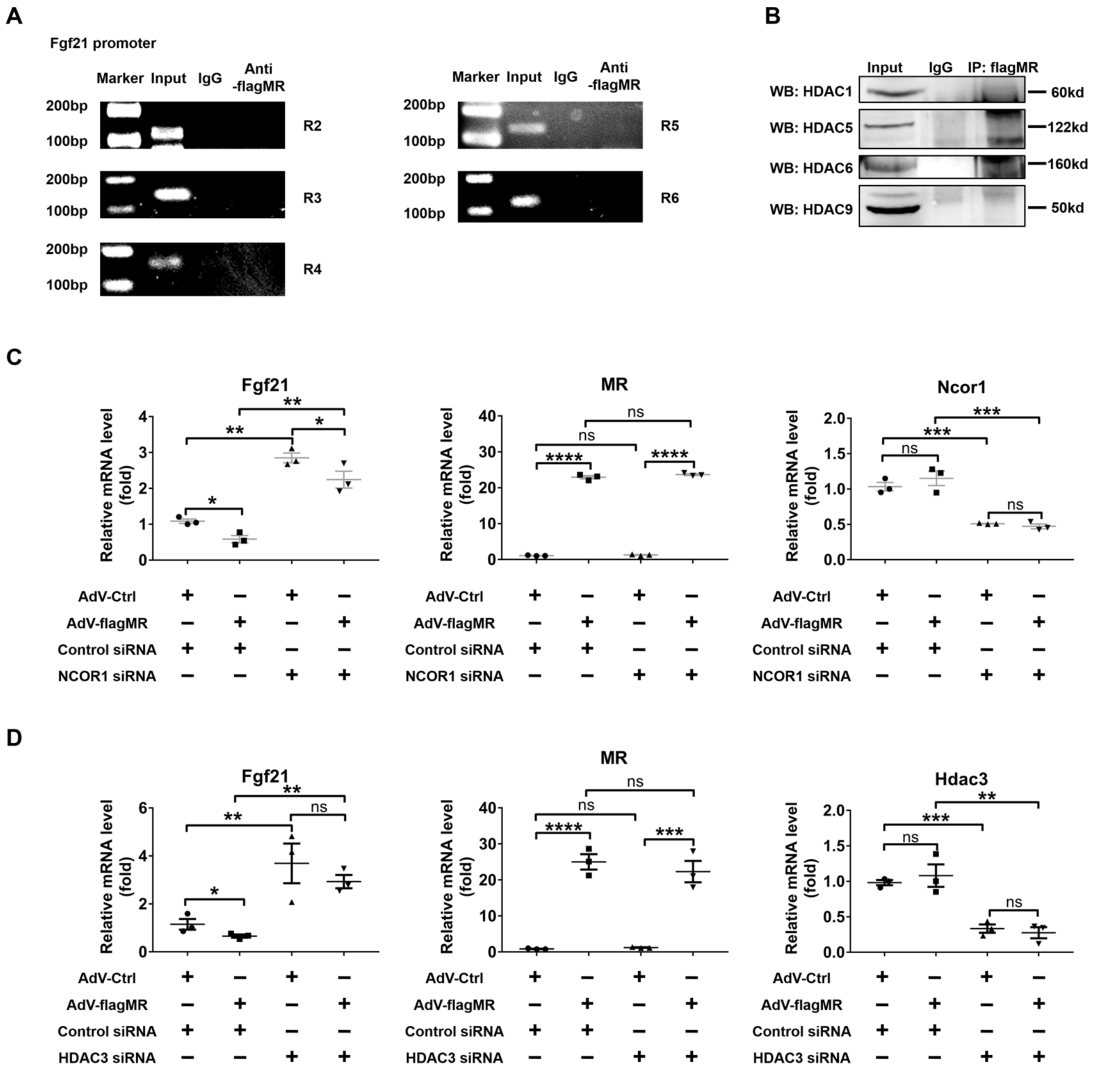

Fig. S4. An MR-NCOR1-HDAC3 complex regulates *Fgf21* expression in hepatocytes. (A) Gel electrophoresis images of regular PCR products using ChIP products pulled down with anti-FLAG antibody in primary hepatocytes. Primers specific to R2-R6 were used for regular PCR. (B) Co-immunoprecipitation analyses of MR and HDACs in primary hepatocytes infected with AdV-flagMR. (C) QRT-PCR analysis of *Fgf21*, *MR* and *NCOR1* gene expression in primary hepatocytes infected with AdV-Ctrl or AdV-flagMR for 24 hours, and then transfected with control siRNA or *NCOR1* siRNA for 48 hours. (D) QRT-PCR analysis of *Fgf21*, *MR* and *HDAC3* gene expression in primary hepatocytes infected with AdV-Ctrl or AdV-flagMR for 24 hours, and then transfected with control siRNA or *HDAC3* siRNA for 48 hours. Data represent three independent experiments. Data are represented as mean ± SEM. ns, not significant. *p<0.05, **p<0.01, ****p<0.0001.

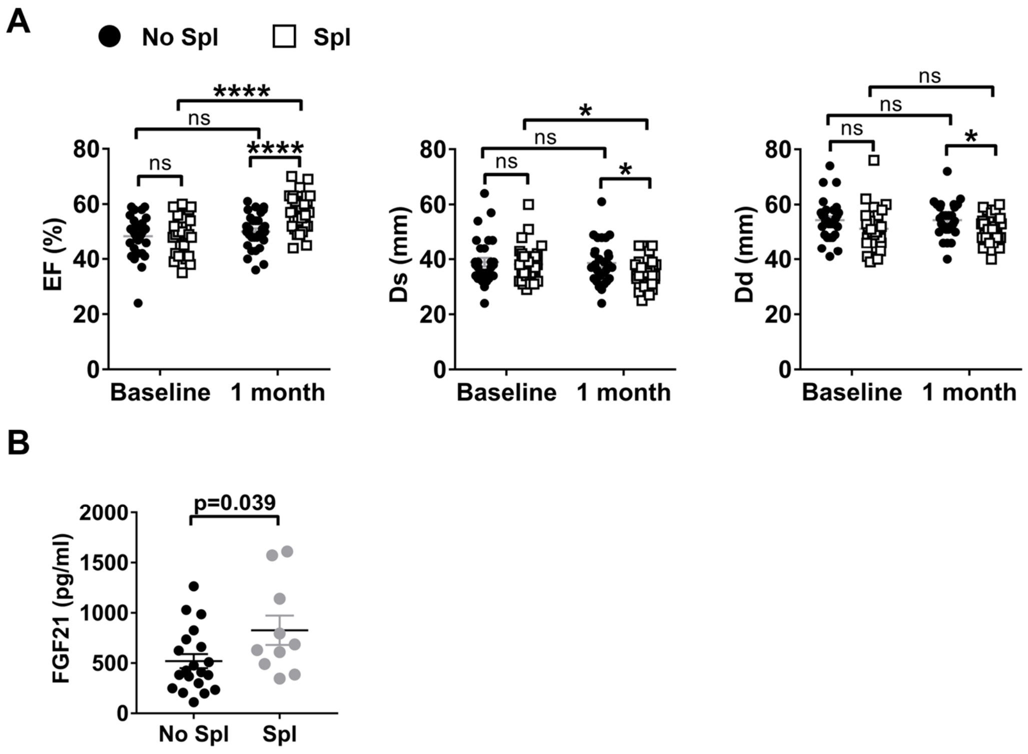

Fig. S5. Spironolactone improves cardiac function and promotes FGF21 secretion in patients. (A) Measurements of LVEF, LVSD and LVDD in HF patients at baseline and 1 month after treatment without or with spironolactone (Spl). n=30. Data are represented as mean ± SEM. ns, not significant. *$p<0.05$, ****$p<0.0001$. (B) Plasma levels of FGF21 tested by ELISA in MI patients 3 days after treatment without or with spironolactone (Spl). n=20:10.

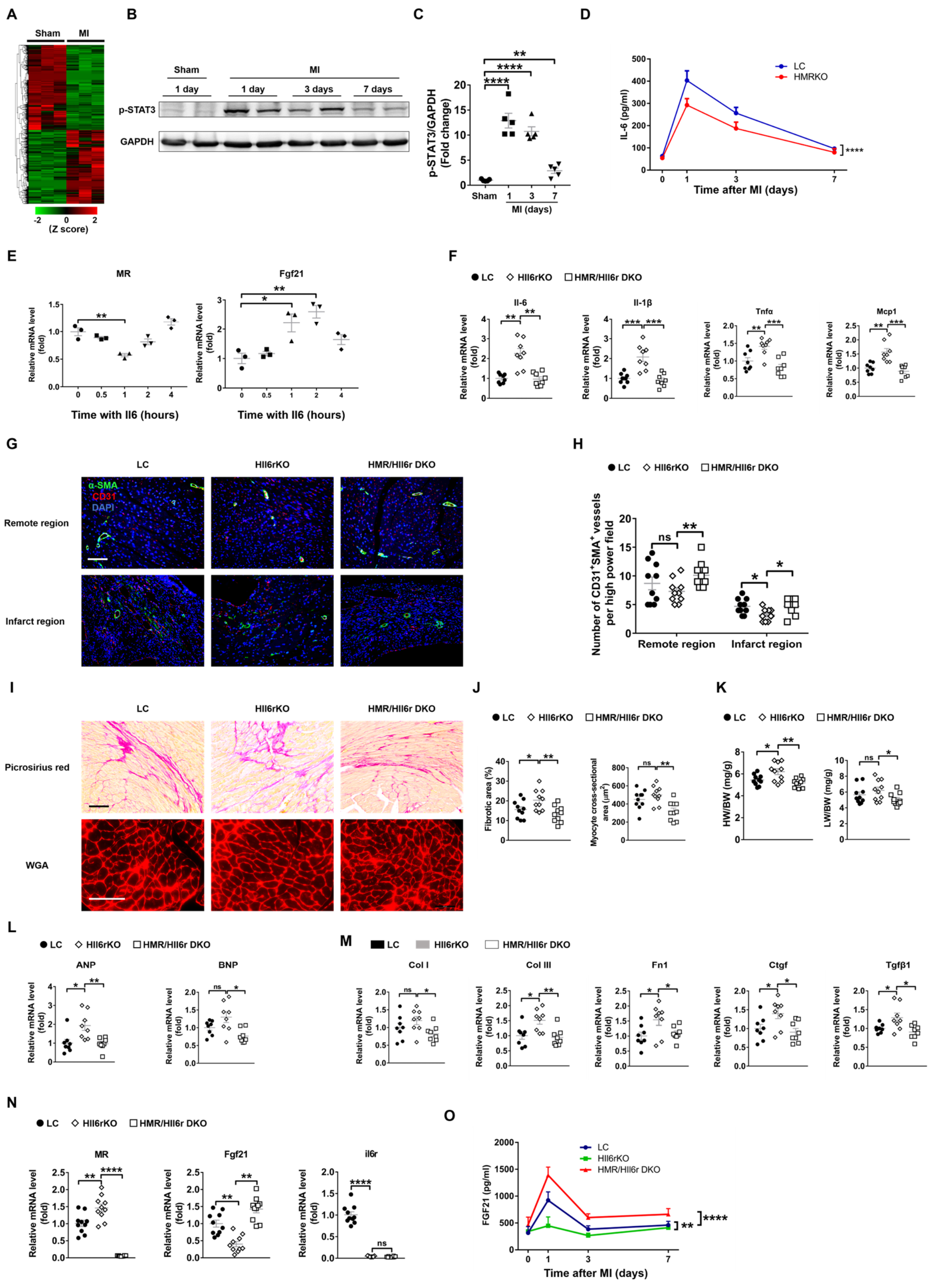

Fig. S6. Hepatic *Il6r* suppresses *MR* expression and exerts cardioprotection after MI in mice. (A) Heatmap of RNA-seq results showing differentially expressed genes in livers 12 hours after MI versus sham operation. n=3. (B) Western blotting analysis of p-STAT3 and GAPDH protein expression in livers of wild type mice at different time points after MI or sham operation. n=5. (C) Quantification of Western blotting results exemplified in (B). (D) Plasma levels of IL6 tested by ELISA in mice at different time points after MI. n=6~8. (E) QRT-PCR analysis of *Fgf21* and *MR* gene expression in primary hepatocytes at different time points after IL6 (20ng/ml) treatment. (F) QRT-PCR analysis of pro-inflammatory cytokines in mouse hearts

3 days after MI. n=8. (G) Representative immunofluorescence staining of α-SMA and CD31 in remote regions and infarct regions of mouse heart samples 7 days after MI. (H) Quantification of neovascularization in remote regions and infarct regions. n=10. (I) Representative Picrosirius red staining and WGA staining of non-infarct regions of mouse heart samples 8 weeks after MI. (J) Quantitative analyses of fibrotic area and cardiomyocyte size. n=10. (K) HW/BW and LW/BW of mice 8 weeks after MI. n=10. (L) QRT-PCR analysis of *ANP* and *BNP* in heart samples 8 weeks after MI. n=8. (M) QRT-PCR analysis of fibrogenic gene expression in heart samples 8 weeks after MI. n=8. (N) QRT-PCR analysis of *MR*, *Il6r* and *Fgf21* gene expression in mouse livers 1 day after MI. n=10. (O) Plasma levels of FGF21 tested by ELISA in mice at different time points after MI. n=6. All scale bars, 100 μm. Data are represented as mean ± SEM. ns, not significant. *p<0.05, **p<0.01, ***p<0.001, ****p<0.0001.

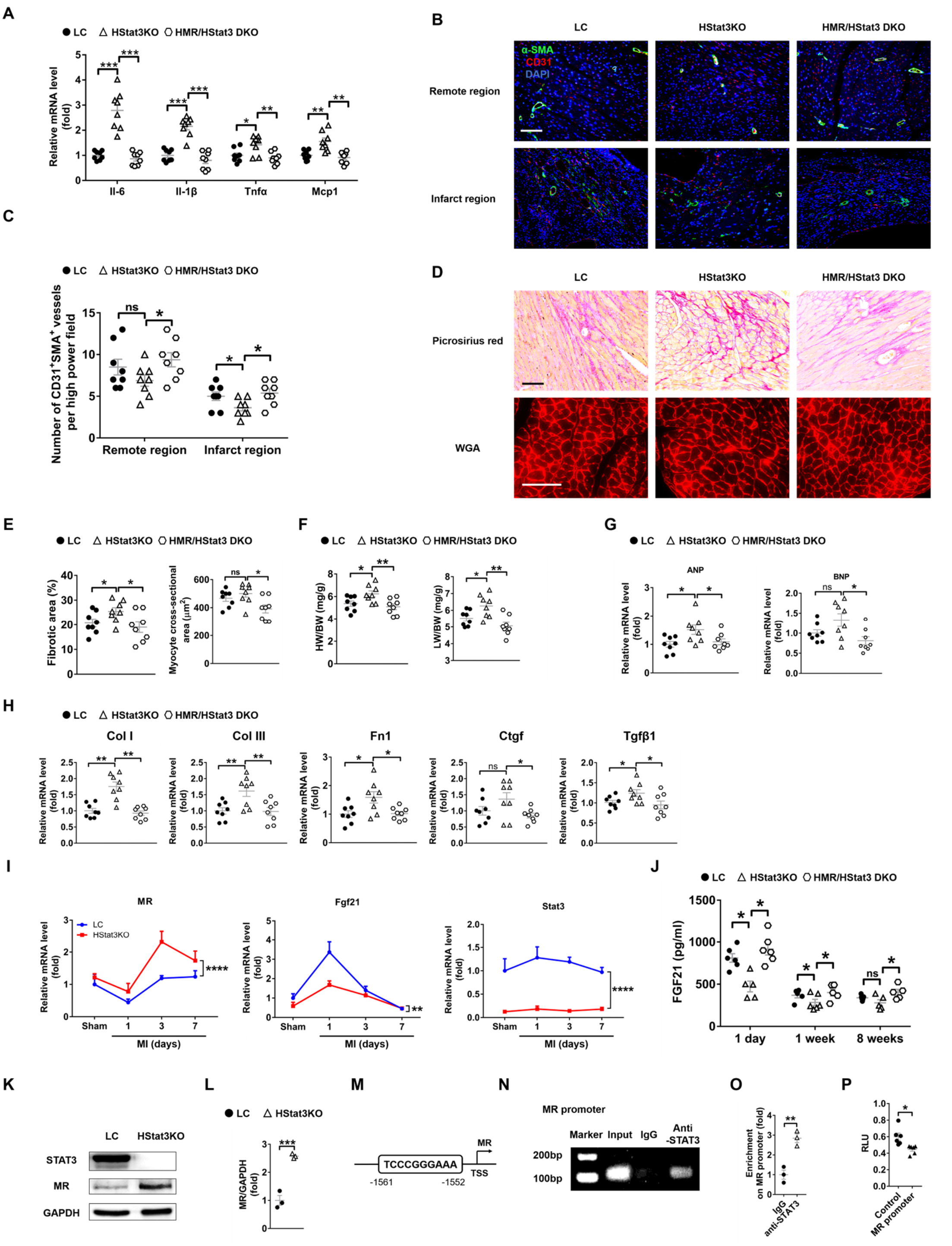

Fig. S7. STAT3 signaling in hepatocytes suppresses *MR* expression and improves cardioprotection after MI in mice. (A) QRT-PCR analysis of pro-inflammatory cytokines in mouse hearts 3 days after MI. n=8. (B) Representative immunofluorescence staining of α-SMA and CD31 in remote regions and infarct regions of mouse heart samples 7 days after MI. (C) Quantification of neovascularization in remote regions and infarct regions. n=8. (D) Representative Picrosirius red staining and WGA staining of non-infarct regions of mouse heart samples 8 weeks after MI. (E) Quantitative analyses of fibrotic area and cardiomyocyte size. n=8. (F) HW/BW and LW/BW of mice 8 weeks after MI. n=8. (G) QRT-PCR analysis of *ANP* and *BNP* in heart samples 8 weeks after MI. n=8. (H) QRT-PCR analysis of fibrogenic gene

expression in heart samples 8 weeks after MI. n=8. (I) QRT-PCR analysis of *Fgf21*, *MR* and *Stat3* gene expression in mouse livers at different time points after MI. n=6. (J) Plasma levels of FGF21 tested by ELISA in mice at different time points after MI. n=5~7. (K) Western blotting analysis of STAT3, MR and GAPDH protein expression in primary hepatocytes from LC and HStat3KO mice. (L) Quantification of (K). (M) Schematic illustration of putative STAT3 binding regions on the promoter of mouse *MR*. (N-O) ChIP analyses showing enrichment of STAT3 on the promoter of *MR* in hepatocytes by regular PCR and gel electrophoresis (N) or QRT-PCR (O). Primers specific to mouse *MR* promoter were used for PCR. (P) Luciferase reporter assays using HEK293FT cells co-transfected with STAT3 plasmid or empty vector (Ctrl) and *MR* promoter plasmid. RLU, relative luciferase unit. Data represent three independent experiments. All scale bars, 100 μm. Data are represented as mean ± SEM. ns, not significant. *$p<0.05$, **$p<0.01$, ***$p<0.001$, ****$p<0.0001$.

Table S1. Baseline Characteristics of Patients with Heart Failure

Characteristics	Spironolactone (n=30)	No spironolactone (n=30)
Age, y	70 ± 9	69 ± 8
Gender, %		
Male	93	90
Female	7	10
Blood pressure, mm Hg		
Systolic	123.9 ± 20.9	125.6 ± 15.38
Diastolic	73.3 ± 10.9	73.4 ± 10.6
Serum potassium concentration, mmol/L	4.0 ± 0.5	4.0 ± 0.4
Serum creatine concentration, μmol/L	92.1 ± 31.5	85.0 ± 24.0
BNP, pg/ml	413.2 ± 490.4	391.2 ± 394.5
Time with heart failure, y	10.9 ± 0.6	11.1 ± 0.5
Medical history, %		
Hypertension	77	76
Arrhythmia	23	20
Diabetes	33	35
Medications, %		
ACEI	73	70
β-blockers	97	93
Diuretics	77	72
Aspirin	97	100
Statins	100	100

Table S2. Baseline Characteristics of Patients with Acute Myocardial Infarction

Characteristics	Spironolactone (n=10)	No spironolactone (n=20)
Age, y	65 ± 4	65 ± 3
Gender, %		
Male	90	80
Female	10	20
Blood pressure, mm Hg		
Systolic	128.8 ± 7.8	132.9 ± 4.7
Diastolic	79.2 ± 4.9	82.5 ± 3.4
Serum potassium concentration, mmol/L	3.7 ± 0.3	3.8 ± 0.1
Serum creatine concentration, μmol/L	120.6 ± 19.4	88.1 ± 8.4
BNP, pg/ml	2470 ± 782.5	1651 ± 581.1
LVEF, %	50.2 ± 3.1	57.2 ± 1.3
Medical history, %		
Hypertension	80	60
Diabetes	40	25
Medications, %		
ACEI/ARB	100	85
β-blockers	100	90
Aspirin	100	100
Statins	90	95

ACEI, angiotensin converting enzyme inhibitor; ARB, angiotensin receptor blocker.

Table S3. Primer Sequences for QRT-PCR

Gene	Forward (5‘-3’)	Reverse (5‘-3’)
MR	TCCAAGATCTGCTTGGTGTGTGGA	AGGCAGGACAGTTCTTTCTCCGAA
Fgf21	CTCTAGGTTTCTTTGCCAACAG	AAGCTGCAGGCCTCAGGAT
Il6	TAGTCCTTCCTACCCCAATTTCC	TTGGTCCTTAGCCACTCCTTC
Il-1β	AAGAGCTTCAGGCAGGCAGTATCA	TGCAGC TGTCTAATGGGA ACGTCA
Tnfα	CCCTCACACTCAGATCATCTTCT	GCTACGACGTGGGCTACAG
Mcp1	CAGCCAGATGCAGTTAACGC	GCCTACTCATTGGGATCATCTTG
ANP	ATTGACAGGATTGGAGCCCAGAGT	TGACACACCACAAGGGCTTAGGAT
BNP	CTCAAGCTGCTTTGGGCACAAGAT	AGCCAGGAGGTCTTCCTACAACAA
Col I	CCGCTGGTCAAGATGGTC	CCTCGCTCTCCAGCCTTT
Col III	TCCTAACCAAGGCTGCAAGA	GGCTGGAAAGAAGTCTGAGGAA
Fn1	CAAGACCATACCTGCCGAATG	GTAGGTGACCCCTCTGGTAAG
Ctgf	GGGCCTCTTCTGCGATTT	ATCCAGGCAAGTGCATTGGTA
Tgfβ1	CAACAATTCCTGGCGTTACCTTGG	GAAAGCCCTGTATTCCGTCTCCTT
Il6r	CCTGAGACTCAAGCAGAAATGG	AGAAGGAAGGTCGGCTTCAGT
Ncor1	CTGGTCTTTCAGCCACCATT	CCTTCATTGGATCCTCCATC
Hdac3	CCGCATCGAGAATCAGAACTC	CCTTGTCGTTGTCATGGTCGCC
Stat3	CAATACCATTGACCTGCCGAT	GAGCGACTCAAACTGCCCT
Gapdh	ATGTTCCAGTATGACTCCACTCACG	GAAGACACCAGTAGACTCCACGACA

Table S4. Primer Sequences for Amplifying Mouse *Fgf21* and *MR* Promoters

Name	5‘-3’
3.2kb *Fgf21*-luc F	GCGTGCTAGCCCGGGCTCGAGCTGGGGGCCTTCTGGGAA
3.2kb *Fgf21*-luc R	ACTTAGATCGCAGATCTCGAGTCATCCATTCCATCAGGGCTG
3.1kb *Fgf21*-luc F	GCGTGCTAGCCCGGGCTCGAGTGGGAGCCAGGGGAAAACCAGATA
3.1kb *Fgf21*-luc R	ACTTAGATCGCAGATCTCGAGTCATCCATTCCATCAGGGCTG
2.8kb *Fgf21*-luc F	GCGTGCTAGCCCGGGCTCGAGTTGCTCTGACAGAAGGGAAGGGAT
2.8kb *Fgf21*-luc R	ACTTAGATCGCAGATCTCGAGTCATCCATTCCATCAGGGCTG
2.2kb *Fgf21*-luc F	GCGTGCTAGCCCGGGCTCGAGGAGACACATCCTACCTGGCGCT
2.2kb *Fgf21*-luc R	ACTTAGATCGCAGATCTCGAGTCATCCATTCCATCAGGGCTG
1.6kb *Fgf21*-luc F	GCGTGCTAGCCCGGGCTCGAGGCCTCTGAAGTGAGGCTGGAGA
1.6kb *Fgf21*-luc R	ACTTAGATCGCAGATCTCGAGTCATCCATTCCATCAGGGCTG
2.0kb *MR*-luc F	GCGTGCTAGCCCGGGCTCGAGCGACAGTGGTGGGAGGGGGC
2.0kb *MR*-luc R	ACTTAGATCGCAGATCTCGAGTGCCGCTCTCCCTGCAGCG

Table S5. Primer Sequences for PCR Analysis of ChIP Products

Name	Forward (5‘-3’)	Reverse (5‘-3’)
Fgf21 Region 1	CTGGGGGCCTTCTGGGAA	CAGGGGCTTGCTTTTACAGTTCTA
Fgf21 Region 2	GTTAAAAAGCCTACTGCAAGTA	ATCCCTTCCCTTCTGTCA
Fgf21 Region 3	TGGCATCCCCAGTGCCAA	AGCGGAGGGCTACGGAGC
Fgf21 Region 4	AAAGTCCGGGGCCCAGCT	GAAGGAACCGTGGGAGAACCTT
Fgf21 Region 5	GAAATTCCACTTGGCTAGC	TGAGACTGGAGCACTGACC
Fgf21 Region 6	AGCACCTTGAAGCTTAAAATT	CTTGGGTCTGAGGGAGGA
MR promoter	GGCGTGATCGATGCTGAG	GGAGCCCCTACACACATGAA
